# Aflatoxin B1 induces subtle but coordinated histone modifications in Epstein-Barr virus infected and non-infected Burkitt lymphoma cells

**DOI:** 10.1016/j.envint.2025.109813

**Published:** 2025-10

**Authors:** Thanos M. Michailidis, Laura Corveleyn, Ruben Almey, Yasmine Bader, Grace A. Odongo, Zdenko Herceg, Rita Khoueiry, Sarah De Saeger, Marthe De Boevre, Maarten Dhaenens

**Affiliations:** a Centre of Excellence in Mycotoxicology and Public Health, Faculty of Pharmaceutical Sciences, Ghent University, Ghent, Belgium; b Epigenomics and Mechanisms Branch, International Agency for Research on Cancer/World Health Organization, Lyon, France; c CRIG, Cancer Research Institute Ghent, Ghent, Belgium; d Laboratory of Pharmaceutical Biotechnology, Faculty of Pharmaceutical Sciences, Ghent University, Gent, Belgium; e Institute of Cancer Research and Genomic Sciences, University of Birmingham, Birmingham, United Kingdom; f Department of Biotechnology and Food Technology, Faculty of Science, University of Johannesburg, Gauteng, South Africa

**Keywords:** Aflatoxin B1, Epstein-Barr virus, Epigenetics, Cancer

## Abstract

Aflatoxin B1 (AFB1), a potent dietary carcinogen, and Epstein-Barr virus (EBV), an oncogenic virus, are both implicated in cancer development, particularly in endemic regions. However, the combined effects of AFB1 exposure and EBV infection on the epigenetic landscape remain poorly understood. This study investigates the impact of AFB1 and EBV on histone post-translational modifications (hPTMs) in Burkitt lymphoma (BL) cells through an integrative approach combining untargeted mass spectrometry-based profiling and a time-lapse experimental design. Our results reveal subtle, yet reproducible and dynamic alterations in histone methylation and acetylation patterns over time in all cells. Specifically, AFB1 exposure induced an increase in H3K27me3 levels, which in the case of EBV infected cells, counteracts the decrease observed at baseline compared to uninfected cells. Additionally, changes in acetylation patterns of H4 N-tail residues and key regulatory proteins suggest potential disruptions in chromatin accessibility and transcriptional regulation. Our correlation and network-based analyses further highlight coordinated epigenetic shifts in response to AFB1, with key acetylation hubs emerging within the histone PTM profile. Despite subtle differences, no significant divergence in overall hPTM responses was detected between EBV-infected and uninfected cells, emphasizing the subtlety of the EBV effect on AFB1 exposure. Future research should explore locus-specific epigenetic changes and therapeutic interventions targeting hPTMs to mitigate cancer risk associated with AFB1 exposure and EBV infection.

## 1 Introduction

Epigenetic regulation is central to cancer development, shaping gene expression and chromatin architecture without altering DNA sequence (Terekhanova et al., 2023). While essential for maintaining cellular identity, these mechanisms are highly susceptible to disruption by environmental exposures (Klibaner-Schiff et al., 2024). Among them, histone post-translational modifications (hPTMs) remain an underexplored yet critical layer of regulation (Audia and Campbell, 2016). These modifications, including acetylation and methylation, are highly dynamic and responsive to external stressors, as shown in recent toxico-epigenetics studies (Verhelst et al., 2022). Disruptions in hPTM patterns can drive tumor progression by altering the expression of genes involved in cell growth, apoptosis, and DNA repair (Millán-Zambrano et al., 2022). Aberrant hPTMs have been implicated in diverse malignancies, including solid tumors and hematological cancers such as non-Hodgkin lymphoma and Burkitt lymphoma (BL) (Bakhshi and Georgel, 2020, Van den Ackerveken et al., 2023).

An endemic form of BL, namely eBL, represents a major pediatric cancer burden in sub-Saharan Africa, where its incidence is among the highest globally (Hämmerl et al., 2019). Epstein-Barr virus (EBV) is a defining etiological factor, with nearly universal infection among affected individuals (Brady et al., 2007, Legason et al., 2023). While MYC translocation is the hallmark genetic driver (Mato et al., 2024), EBV reshapes the epigenome of infected B cells, altering chromatin accessibility and transcriptional programs to promote survival and proliferation (Scott, 2017). The virus induces histone modifications and DNA methylation changes that sustain oncogenic signaling and facilitate immune evasion (Ghosh Roy et al., 2016). However, reports on EBV-driven epigenetic reprogramming remain highly fragmented (Gao et al., 2024).

Several environmental factors, including dietary carcinogens and endemic oncogenic viruses, have been demonstrated to interact and contribute to carcinogenesis (Herceg, 2007, Herceg et al., 2018). In sub-Saharan Africa, where eBL is most prevalent, chronic exposure to mycotoxins such as aflatoxin B1 (AFB1) is widespread. Classified as a Group 1 carcinogen by the International Agency for Research on Cancer (IARC), AFB1 is best known for its synergy with hepatitis B virus (HBV) in hepatocellular carcinoma (HCC), where it promotes mutagenesis and epigenetic alterations (IARC., 2012, Wild and Montesano, 2009). Recent evidence suggests that similar interactions may extend to other viral-associated malignancies, raising the question of whether AFB1 influences the epigenetic landscape of EBV-infected B cells (Mouchtaris Michailidis et al., 2024).

AFB1 has primarily been studied for its effects on DNA methylation, where it regulates tumor suppressor genes in HCC and influences immune-related gene expression (Herceg et al., 2012, Hernandez-Vargas et al., 2015). Early findings suggested an interaction with the histone code (Groopman et al., 1980), yet most subsequent research has focused on its role in reproductive toxicity (Liu et al., 2015, Zhu et al., 2014). In HCC models, AFB1 exposure has been associated with increased histone deacetylation, leading to chromatin compaction and transcriptional repression (Rieswijk et al., 2016). Emerging data also suggest an epigenetic interplay between AFB1 exposure and EBV infection in eBL, expanding the scope of environmental contributions to lymphomagenesis. AFB1 has been reported to induce the (re)activation of the EBV lytic cycle and increase viral load through the activation of signaling pathways such as PI3K (Accardi et al., 2015, Maroui et al., 2024). Additionally, both AFB1 and EBV drive epigenetic modifications in B cells that may contribute to disease progression, including DNA methylation changes in key regulatory genes such as the tumor suppressor *TGFBI* (Manara et al., 2022). Whether AFB1 exacerbates EBV-mediated chromatin remodeling or independently alters histone landscapes in infected B cells remains an open question, underscoring the need to further investigate the epigenetic mechanisms underlying this interaction.

Here, we present a comprehensive, untargeted mass spectrometry-based analysis of hPTMs and co-extracted proteins in BL cells with and without EBV infection following temporal exposure to AFB1. Our findings reveal subtle but significant changes in the histone and proteome landscape, alongside coordinated alterations in histone mark interconnectivity. By mapping these hPTM dynamics in response to viral and environmental exposures, this study provides new insights into the epigenetic consequences associated with AFB1 in BL, offering a framework to further investigate how these modifications intersect with EBV-driven mechanisms.

## 2 Methods

### 2.1 Cell culture

Two BL cell lines (Louckes) were provided by the IARC biobank. The one is infected with EBV (EBV+) and the other is not infected (EBV−). The EBV infection protocol was an adaptation from Lopez-Serra P., and Esteller M., 2011 (Lopez-Serra and Esteller, 2011). The cell lines were cultured in RPMI 1640 media (Gibco™ 21870076, Thermo Fisher Scientific), enriched with 10 % Fetal Bovine Serum (Gibco™ 16250078, Thermo Fisher Scientific), 1 % Penicillin/Streptomycin (100 U/ml / 100 mg/ml, Gibco™ 15140122, Thermo Fisher Scientific), 1 mM Sodium Pyruvate (Gibco™ 11360070, Thermo Fisher Scientific), and 2 mM L-glutamine (Gibco™ 25030081, Thermo Fisher Scientific). The cells were maintained at 37 °C, 95 % humidity, and 5 % CO2, with passaging every 48 h.

### 2.2 AFB1 exposure and sample collection

A cytotoxicity test utilizing the MTS (3-(4,5-dimethylthiazol-2-yl)-5-(3-carboxymethoxyphenyl)-2-(4-sulfophenyl)–2H-tetrazolium) assay (G3580, Promega, CellTiter96 Aqueous One Solution Cell Proliferation Assay) was first conducted to determine the appropriate concentration of AFB1 (Maroui et al., 2024). Various concentrations were tested over 24, 48, and 72 h with the highest maintaining above 80 % viability (O.D fold control above 0.8) being selected for experimental exposures. The cells were then exposed to 50 µM AFB1 (A6636-5MG Sigma Aldrich). Dimethyl sulfoxide (DMSO, 472301-100ML Sigma Aldrich) served as the solvent control at 0.1 %. Valproic acid (VPA) (P4543-100G Sigma Aldrich) 1 mM was used as a positive control after 48 h of exposure. The cells were exposed to AFB1 for 24, 48 and 72 h. Post-exposure, cell viability was assessed prior to cell harvesting. Cells were harvested by centrifugation (5  min, 1000 RPM, 4 °C) at a concentration of 2*10^6^ cells/mL. After washing the cell pellets with 1  mL cold PBS, the cell pellets were snap-frozen in −150 °C and stored in −80 °C. For each cell line, 6 biological replicates were harvested at each different time point.

### 2.3 Histone sample preparation

Histone extraction of the cells was performed using direct acid extraction as described previously (Govaert et al., 2016, Verhelst and Corveleyn, 2024). Consequently, one-dimensional SDS-PAGE (sodium dodecyl sulfate–polyacrylamide gel electrophoresis) on a 9–18 % TGX gel (Bio-Rad) was performed on a fraction of the resulting extracts for quantification and normalization (Verhelst, 2024). The remaining histone extracts were propionylated and digested following an established protocol (Corveleyn and Verhelst, 2024, Meert et al., 2016).

### 2.4 Liquid chromatography and mass spectrometry acquisition

The propionylated and digested samples were resuspended in 0.1 % formic acid and injection volumes were adjusted resulting in 800 ng of histones on column. A quality control mixture was created by mixing 2  μL of each sample. Data-dependent acquisition (DDA) was performed on a ZenoTOF 7600 system (AB Sciex) operating in positive mode electrospray ionization (ESI) coupled to an ACQUITY UPLC M−Class System (Waters) operating in capillary flow mode (5  μL/min). Trapping and separation of the peptides was carried out on a Triart C18 column (5  mm × 0.5  mm; YMC) and a YMC-Triart C18 column at 45 °C (150 mm × 0.3  mm, particle size 3  µm), respectively, using a low pH reverse-phase gradient. Buffers A and B of the mobile phase consisted of 0.1 % formic acid in water and in acetonitrile, respectively. A 20-min non-linear gradient going from 2 % to 50 % Buffer B, followed by a washing step at 90 % mobile phase B and an equilibration step at 2 % mobile phase B (starting conditions). The samples were run in a randomized fashion and a quality control injection was incorporated every five samples. For each cycle, one full MS1 scan (*m*/*z* 350–1,250) of 100  ms was followed by an MS2 (*m*/*z* 140–1,800) of 12  ms, resulting in a maximum cycle time of 800 ms. A maximum of 40 precursors (charge state +2 to +6) exceeding 1500 c.p.s. were monitored, followed by an exclusion for 3  s per cycle. An MS1 collision energy of 12  V and dynamic MS2 collision energy was applied. Ion source parameters were set to 4.5  kV for the ion spray voltage, 35 psi for the curtain gas, 15 psi for nebulizer gas (ion source gas 1), 60 psi for heater gas (ion source gas 2) and 200 °C as source temperature.

### 2.5 Data analysis

LC–MS data analysis was performed using Progenesis QIP (v4.2) and Mascot (v2.8.3) as previously described (Verhelst et al., 2020). In addition to calculating relative abundance metrics however, single hPTM abundances were inferred according to the msqrob2PTM workflow (Demeulemeester et al., 2024). Briefly, peptide-level data is normalized followed by robust summarization to either the protein or single hPTM level (Sticker et al., 2020). A statistical model describing the experiment is then fit for every measured hPTM/peptidoform/protein abundance, where msqrob2 allows for robust fitting of linear mixed models. Of note, due to limitations in fragmentation spectra, H3K36 and K37 are often indistinguishable in mass spectrometry, as the diagnostic fragment ion that separates them is rarely formed. The Mascot search engine therefore reports both residues in the result file, and we maintained this annotation throughout for consistency. However, for biological interpretation, readers should consider the signal as representing H3K36.

Estimated model coefficients are compared through the relevant contrasts and Benjamini–Hochberg adjusted p-values are calculated for each resulting *t*-test statistic. The full code of the statistical analysis is available in Supplementary Data 1. Functional enrichment and protein interaction analysis of the acid extractome was conducted using String-DB (v12.0) and further functional annotation of individual proteins was carried out in UniProt.

## 3 Results

### 3.1 Experimental design

The study was designed to capture hPTM responses to toxicological exposure in both infected and non-infected cells, building on recent findings that such changes can occur rapidly post-incubation (Verhelst et al., 2022). VPA, a well-documented histone deacetylase inhibitor (Ni et al., 2017), served as the positive control, providing a benchmark for assessing the epigenetic alterations induced by AFB1.

Louckes cells, both EBV-negative (EBV−) and EBV-positive (EBV+) were treated with AFB1, DMSO, and VPA, across a temporal spectrum of 0 h to 72 h. Following treatment, cells underwent a standardized histone extraction protocol in three randomized batches. The isolated histones were then propionylated, digested, and subjected to a secondary propionylation and reversal of nonspecific derivatization. Through this chemical derivatization, monomethylated lysines are converted into butyrylated lysines and they are therefore depicted as such in the downstream analysis. After SDS-PAGE normalization, liquid chromatography-tandem mass spectrometry (LC-MS/MS) was employed. All sample preparation and measurements were conducted in a randomized fashion to minimize batch effects for the 120-sample batch. A pooled quality control (QC) sample, incorporating all conditions, was analyzed every five runs to monitor instrument stability.

**Fig. 1:**
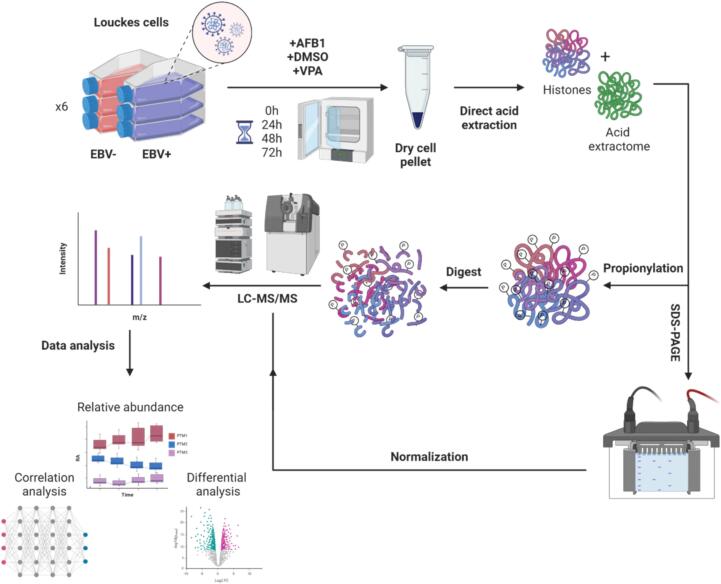
Schematic overview of the experimental design for investigating the impact of EBV infection and AFB1 exposure on histone post-translational modifications (hPTMs). Louckes cells, both EBV-negative (EBV−) and EBV-positive (EBV+), were cultured and treated with AFB1, DMSO, or VPA at multiple time points (0 h, 24 h, 48 h, 72 h). Following treatment, cells were collected as dry pellets and subjected to direct acid extraction to obtain histones and the acid extractome. Extracted histones were processed via SDS-PAGE for protein separation, followed by propionylation and enzymatic digestion. The processed samples were analyzed using liquid chromatography-tandem mass spectrometry (LC-MS/MS) to determine the relative abundance of hPTMs and quantify co-extracted proteins. Normalized data were subjected to downstream analyses, including correlation analysis and differential analysis, to assess the impact of treatments on histone modifications over time.

Quantitative data analysis of the histone code can be expressed at two different levels: (i) at the level of peptidoforms, which are the measured peptides including combinatorial PTMs, and (ii) at the level of single hPTMs by inferring either relative abundance (Sidoli et al., 2016) or the “PTM usage” as described in the msqrob2PTM workflow (Demeulemeester et al., 2024), which was used in this study. Notably, PTM usage includes unmodified residues (Unmod) as a functional PTM, given that many chromatin-binding proteins prefer the unmodified sites — for example, DNMT3a and DNMT3b use their ADD domain to selectively bind H3K4Unmod. Finally, over 1000 proteins are co-extracted with the histones, collectively termed the acid extractome, a proteomic subfraction enriched in basic, nucleic acid-binding proteins (De Clerck et al., 2019, Zijlmans et al., 2022). Fig. 1 provides an at-a-glance view of the entire experimental workflow, delineating each step from cell culture to data analysis.

### 3.2 Experimental validation

**Fig. 2:**
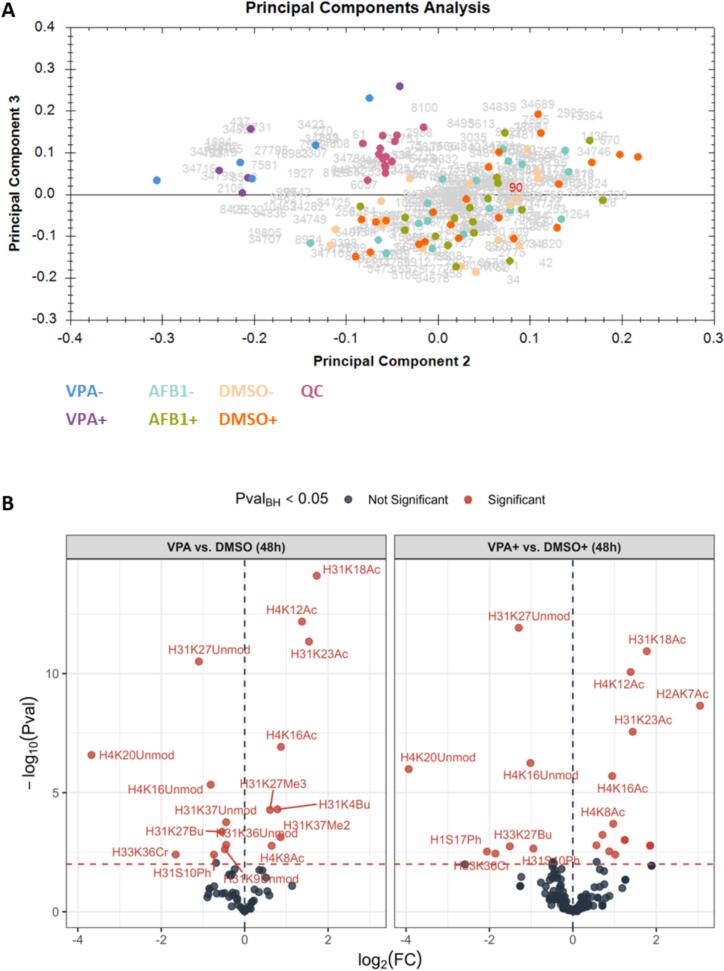
Method Validation and Data Quality Control. (A) Principal component analysis (PCA) of all measured histone peptidoforms along PC2 and PC3 shows central clustering of quality control (QC) samples (yellow) and distinct separation of 1 mM valproic acid (VPA)-treated samples (red) toward the upper left quadrant. (B) Volcano plots depicting significant changes in histone modifications after 48 h of VPA exposure compared to dimethyl sulfoxide (DMSO) controls. The left panel represents uninfected cells, while the right panel shows Epstein-Barr virus (EBV)-infected cells. *Note: Butyrylated (bu) residues should be interpreted as methylated (me) sites due to sample preparation, as described in the Methods.* (For interpretation of the references to color in this figure legend, the reader is referred to the web version of this article.)

Principal Component Analysis (PCA) (Fig. 2A) of all measured histone peptidoforms provides an initial indicator of the data quality and global trends in histone modification patterns. The central clustering of QC samples confirms instrument stability, while VPA-treated samples cluster distinctly in the upper left quadrant, indicating a consistent response across both cell cultures. From a biological perspective, this clustering underscores that both infected and uninfected cell lines remain responsive in terms of acetylations, a proxy for more general epigenetic alterations on histones and an essential prerequisite for evaluating the impact of treatments on the histone code.

While peptidoforms reflect the actual measurement by the instrument, single hPTMs need to be inferred at the data level for biological interpretation. Using msqrob2PTM (Demeulemeester et al., 2024), we observe a significant increase in single acetylations on histones H3 and H4 following 48 h of VPA treatment in both EBV− and EBV+ Louckes cells compared to DMSO controls. The most pronounced increases were observed at H3K18-K23 and H4K12-K16 (Fig. 2B). Note that such changes usually are accompanied by a decrease in the original PTMs on these respective residues, which for VPA are mainly unmodified lysines. These findings align with established effects of VPA on histone acetylation previously reported in various disease models and cellular environments (Verhelst et al., 2020, Verhelst et al., 2022) reinforcing the experimental framework. Notably, both EBV− and EBV+ cells exhibited robust and nearly identical responses, not only highlighting the quantitative reliability of the dataset but also confirming that EBV infection does not impair epigenetic responsiveness.

### 3.3 Data modeling

An interactive, comprehensive data analysis report is provided in Supplementary Data 1. Analysis of all 4,623 peptides from the combined histone and acid extractome dataset (Supplementary Fig. 1A) revealed a batch effect across the three independently processed batches. Specifically, the histone-to-co-extracted peptide ratio varied significantly, leading to batch-specific clustering in scatter and multidimensional scaling (MDS) plots (Supplementary Fig. 1B and C). To correct for this, data normalization was performed in two steps: (i) variance stabilization (VSN) (Huber et al., 2002) to remove technical variation, such as sample loading differences; and (ii) adjustment for nucleosome abundance differences by centering histone PTM fold changes across samples. This approach, similar to Koopmans et al. 2023 (Koopmans et al., 2023) was performed at the hPTM level to ensure a centered distribution of fold changes (Supplementary Fig. 2A and B), defining a total of 146 single hPTMs for differential analysis (Supplementary Fig. 2C). Additionally, intra-block correlation estimates confirmed the need to account for sample batches as random effects, with correlation values around 0.4, underscoring the necessity of batch correction to improve downstream statistical robustness.

To disentangle the different contributions of EBV infection, AFB1 treatment, and time, we fitted two models: one “means model” with time as a factor and one “regression model” with linear time as a covariate (Supplementary Fig. 3). Briefly, the “means model” allows testing for hPTMs and co-extracted proteins that are differentially up- or downregulated between every sample group of the design. While intuitive and powerful for this purpose, these comparisons cannot completely capture temporal differences between given treatments or infection status. Although it decreases the interpretability, the “regression model” answers these time-responsive differential questions directly, which is very different from comparing feature abundances in a binary comparison. Therefore, the main focus will be on these time regression models.

### 3.4 The effect of EBV infection on Louckes cells at baseline

**Fig. 3:**
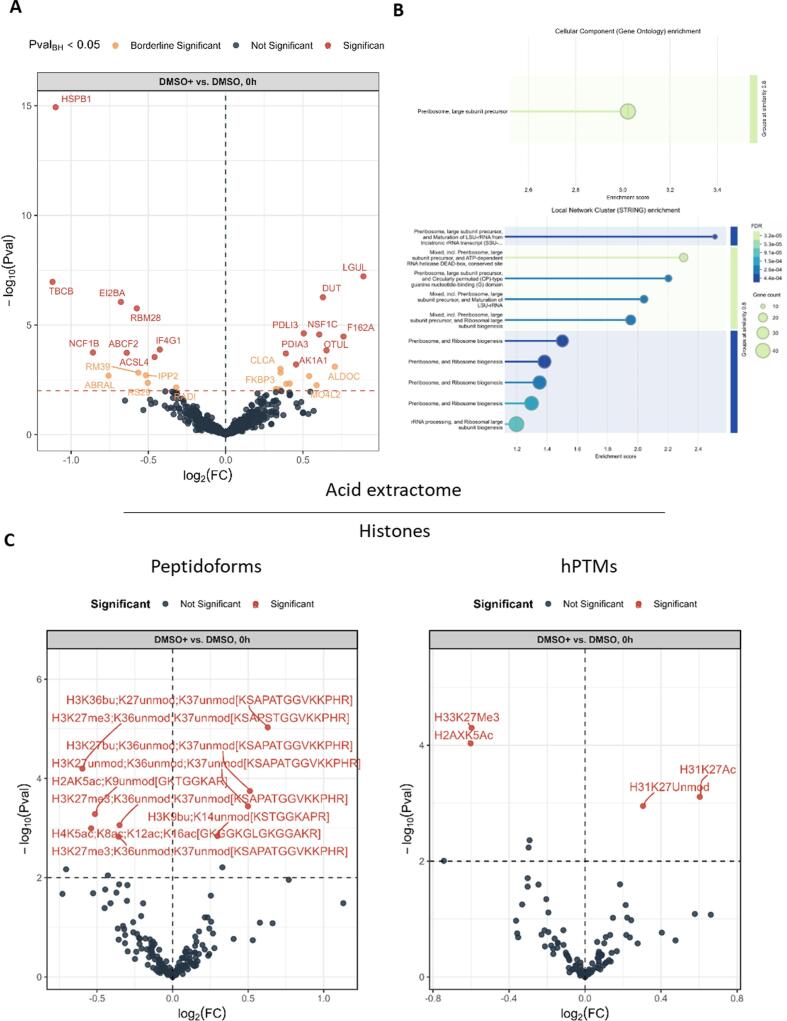
The impact of EBV on the histone code at baseline. (A) Volcano plot showing differentially abundant co-extracted proteins between EBV-infected (DMSO+) and uninfected (DMSO) Louckes cells treated with DMSO. (B) Gene set enrichment analysis (GSEA). Top: Significantly enriched Gene Ontology (GO) term 0030687. Bottom: Local network clustering of functionally related proteins, where bubble size represents gene count and color intensity reflects statistical significance (false discovery rate, FDR). Horizontal lines indicate protein–protein interactions. (C) Impact of EBV infection on histone modifications. The left panel displays measured peptidoform abundances, while the right panel presents inferred hPTMs. EBV infection is associated with changes in the H3K27–R40 region, including a reduction in H3K27me3. *Note: Butyrylated (bu) residues should be interpreted as methylated (me) sites due to sample preparation, as described in the Methods.*

To assess the impact of EBV infection on the cell line at the onset, i.e. baseline, of the experiment, we first analyzed the protein fraction that was co-extracted with the histones, known as the acid extractome. This so-called acid extractome contains a lot of valuable information on the cellular phenotype at baseline before incubation with AFB1. At the start of the experiment, a few proteins were differentially expressed between EBV-infected and uninfected cells (Fig. 3A). While the number of differentially expressed proteins was insufficient for a statistical gene ontology (GO) analysis, Table 1 summarizes the individual functions of significantly differential proteins. Notably, three upregulated proteins in the infected cells are involved in immune response (NF-kappa-B activity and MHC), while others are involved in metabolic detoxification, apoptosis, actin cytoskeleton, transitional endoplasmic reticulum and dUTP −> dUMP conversion (Table 1). Gene set enrichment analysis (GSEA) of protein fold changes revealed a single significantly enriched term, pre-ribosome large subunit precursor (GO:0030687) in EBV-negative cells (Fig. 3B). In eukaryotes, ribosome assembly is a rate-limiting step in ribosomal biogenesis that takes place in a distinctive subnuclear organelle, the nucleolus (Abetov et al., 2019). This implies that the (pre)ribosomal biogenesis in infected cells is reduced. Of note, database searches including the EBV proteome did not yield any identifications of the viral proteome and no differential proteins were detected between AFB1 and DMSO at 0 h in infected or uninfected Louckes, as expected.

**Table 1:** Differentially expressed proteins across all comparisons with their UniProt functional annotation.

Gene name	Function (UniProt)	Fold-change	q-Value	Comparison
HSPB1	Molecular chaperone probably maintaining denatured proteins in a folding-competent state. Plays a role in stress resistance and actin organization	−1.1	1.26E-12	DMSO+ vs. DMSO, 0 h
LGUL	Catalyzes the conversion of hemimercaptal, formed from methylglyoxal and glutathione, to S-lactoylglutathione. Involved in the regulation of TNF-induced transcriptional activity of NF-kappa-B	0.891	3.29E-05	DMSO+ vs. DMSO, 0 h
TBCB	Involved in regulation of tubulin heterodimer dissociation. May function as a negative regulator of axonal growth	−1.12	3.90E-05	DMSO+ vs. DMSO, 0 h
DUT	Inhibits peroxisome proliferator-activated receptor (PPAR) activity by binding of its N-terminal to PPAR, preventing the latter's dimerization with retinoid X receptor	0.629	0.000148	DMSO+ vs. DMSO, 0 h
EI2BA	Acts as a component of the translation initiation factor 2B (eIF2B) complex, which catalyzes the exchange of GDP for GTP on eukaryotic initiation factor 2 (eIF2) gamma subunit	−0.678	0.000194	DMSO+ vs. DMSO, 0 h
RBM28	Nucleolar component of the spliceosomal ribonucleoprotein complexes	−0.575	0.000317	DMSO+ vs. DMSO, 0 h
PDLI3	May play a role in the organization of actin filament arrays within muscle cells	0.505	0.00369	DMSO+ vs. DMSO, 0 h
NSF1C	Reduces the ATPase activity of VCP	0.605	0.00369	DMSO+ vs. DMSO, 0 h
F162A	May be involved in hypoxia-induced cell death of transformed cells	0.762	0.00406	DMSO+ vs. DMSO, 0 h
IF4G1	Component of the protein complex eIF4F, which is involved in the recognition of the mRNA cap, ATP-dependent unwinding of 5′-terminal secondary structure and recruitment of mRNA to the ribosome	−0.426	0.0142	DMSO+ vs. DMSO, 0 h
OTUL	Deubiquitinase that specifically removes linear ('Met-1′-linked) polyubiquitin chains to substrates and acts as a regulator of angiogenesis and innate immune response	0.652	0.0142	DMSO+ vs. DMSO, 0 h
NCF1B	May be required for activation of the latent NADPH oxidase (necessary for superoxide production)	−0.859	0.0154	DMSO+ vs. DMSO, 0 h
ABCF2	Lacks transmembrane domains and is probably not involved in transport	−0.64	0.0154	DMSO+ vs. DMSO, 0 h
PDIA3	Protein disulfide isomerase that catalyzes the formation, isomerization, and reduction or oxidation of disulfide bonds in client proteins and functions as a protein folding chaperone	0.389	0.0154	DMSO+ vs. DMSO, 0 h
ACSL4	Catalyzes the conversion of long-chain fatty acids to their active form acyl-CoA for both synthesis of cellular lipids, and degradation via beta-oxidation	−0.46	0.0209	DMSO+ vs. DMSO, 0 h
AK1A1	Catalyzes the NADPH-dependent reduction of a wide variety of carbonyl-containing compounds to their corresponding alcohols	0.455	0.0429	DMSO+ vs. DMSO, 0 h
LAP2B	May help direct the assembly of the nuclear lamina and thereby help maintain the structural organization of the nuclear envelope	−3.14	0.00261	DMSO+ vs. DMSO linear time effect
ITF2	Transcription factor that binds to the immunoglobulin enhancer Mu-E5/KE5-motif. Involved in the initiation of neuronal differentiation	2.71	0.00261	DMSO+ vs. DMSO linear time effect
RM39	/	3.4	0.017	DMSO+ vs. DMSO linear time effect
VIME	Plays a role in cell directional movement, orientation, cell sheet organization and Golgi complex polarization at the cell migration front	0.00836	2.28E-08	AFB1+ vs. DMSO+ linear time effect
OBF1	Transcriptional coactivator that specifically associates with either POU2F1/OCT1 or POU2F2/OCT2	−0.00796	0.000519	AFB1+ vs. DMSO+ linear time effect
IGHM	Serves as receptor which, upon binding of a specific antigen, triggers the clonal expansion and differentiation of B lymphocytes into immunoglobulins-secreting plasma cells	−0.00738	0.0233	AFB1+ vs. DMSO+ linear time effect
ASNS	Catalytic activity of L-aspartate	−0.00737	0.0233	AFB1+ vs. DMSO+ linear time effect
RBM34	/	−0.00657	0.029	AFB1+ vs. DMSO+ linear time effect
EBP2	Required for the processing of the 27S pre-rRNA	−0.00479	0.0319	AFB1+ vs. DMSO+ linear time effect
VIME	Plays a role in cell directional movement, orientation, cell sheet organization and Golgi complex polarization at the cell migration front	0.481	0.000227	AFB1+ vs DMSO+, 72 h
OBF1	Transcriptional coactivator that specifically associates with either POU2F1/OCT1 or POU2F2/OCT2	−0.514	0.00286	AFB1+ vs DMSO+, 72 h
VIME	Plays a role in cell directional movement, orientation, cell sheet organization and Golgi complex polarization at the cell migration front	0.586	7.03E-05	AFB1+ vs DMSO+, 48 h
DNJB1	Interacts with HSP70 and can stimulate its ATPase activity	−0.00645	5.78E-05	AFB1 vs. DMSO linear time effect
RL1D1	Regulates cellular senescence through inhibition of PTEN translation. Acts as a pro-apoptotic regulator in response to DNA damage	−0.00705	8.48E-05	AFB1 vs. DMSO linear time effect
HMGCL	Mitochondrial 3-hydroxy-3-methylglutaryl-CoA lyase that catalyzes a cation-dependent cleavage of (S)-3-hydroxy-3-methylglutaryl-CoA into acetyl-CoA and acetoacetate, a key step in ketogenesis	0.0107	0.00105	AFB1 vs. DMSO linear time effect
ECHB	Mitochondrial trifunctional enzyme catalyzes the last three of the four reactions of the mitochondrial beta-oxidation pathway	0.00767	0.00175	AFB1 vs. DMSO linear time effect
RBM34	/	−0.00827	0.00175	AFB1 vs. DMSO linear time effect
EBP2	Required for the processing of the 27S pre-rRNA	−0.00611	0.00178	AFB1 vs. DMSO linear time effect
VIME	Plays a role in cell directional movement, orientation, cell sheet organization and Golgi complex polarization at the cell migration front	0.00531	0.0021	AFB1 vs. DMSO linear time effect
IGHM	Serves as receptor which, upon binding of a specific antigen, triggers the clonal expansion and differentiation of B lymphocytes into immunoglobulins-secreting plasma cells	−0.00746	0.0165	AFB1 vs. DMSO linear time effect
UCHL3	Deubiquitinating enzyme (DUB) that controls levels of cellular ubiquitin through processing of ubiquitin precursors and ubiquitinated proteins	0.0045	0.0165	AFB1 vs. DMSO linear time effect
CF298	Plays a role in motile cilium function, possibly by acting on outer dynein arm assembly	−0.00793	0.0165	AFB1 vs. DMSO linear time effect
RBM28	Nucleolar component of the spliceosomal ribonucleoprotein complexes	−0.00455	0.041	AFB1 vs. DMSO linear time effect
VINC	Actin filament (F-actin)-binding protein involved in cell-matrix adhesion and cell–cell adhesion	0.00362	0.041	AFB1 vs. DMSO linear time effect
OBF1	Transcriptional coactivator that specifically associates with either POU2F1/OCT1 or POU2F2/OCT2	−0.00599	0.0415	AFB1 vs. DMSO linear time effect
DDX46	Component of the 17S U2 SnRNP complex of the spliceosome, a large ribonucleoprotein complex that removes introns from transcribed pre-mRNAs	−0.00708	0.0464	AFB1 vs. DMSO linear time effect
RL1D1	Regulates cellular senescence through inhibition of PTEN translation	−0.522	0.00234	AFB1 vs DMSO, 48 h
IGHM	Serves as receptor which, upon binding of a specific antigen, triggers the clonal expansion and differentiation of B lymphocytes into immunoglobulins-secreting plasma cells	−0.611	0.00594	AFB1 vs DMSO, 48 h
HMGCL	Mitochondrial 3-hydroxy-3-methylglutaryl-CoA lyase that catalyzes a cation-dependent cleavage of (S)-3-hydroxy-3-methylglutaryl-CoA into acetyl-CoA and acetoacetate, a key step in ketogenesis	0.641	0.0159	AFB1 vs DMSO, 48 h
DNJB1	Interacts with HSP70 and can stimulate its ATPase activity	−0.378	0.0159	AFB1 vs DMSO, 48 h
RIR2	Provides the precursors necessary for DNA synthesis. Catalyzes the biosynthesis of deoxyribonucleotides from the corresponding ribonucleotides. Inhibits Wnt signaling	0.686	0.027	AFB1 vs DMSO, 48 h
OBF1	Transcriptional coactivator that specifically associates with either POU2F1/OCT1 or POU2F2/OCT2	−0.66	0.000729	AFB1 vs DMSO, 24 h

Fig. 3C shows the impact of EBV infection on the histone code at the onset of the experiment, showing both the measured peptidoform and the inferred single PTM abundance at baseline. The most striking significant changes occur on the H3K27-R40 peptide stretch, where EBV infection leads to a reduction in H33K27me3, accompanied by an increase in acetylated and unmodified H31K27. While EBV infection is known to influence H3K27ac levels (Zhou et al., 2015), our analysis reveals that this increase occurs at the expense of H33K27me3 (Fig. 3C). Importantly, single PTMs like H3K27ac can be significantly regulated even if no individual combinatorially modified peptidoform meets the statistical significance threshold. Given the central role of H3K27me3 in numerous cellular processes, including its function as a gatekeeper of pluripotency in mouse and human embryonic stem cells (De Clerck et al., 2019, van Mierlo et al., 2019, Zijlmans et al., 2022), its downregulation may have broader epigenetic consequences. Additionally, EBV infection leads to reduced H2AK5 acetylation, a modification associated with the DNA damage response (Ikura et al., 2015), highlighting key baseline epigenetic differences prior to AFB1 exposure.

### 3.5 AFB1 induces subtle differences in protein expression through time

**Fig. 4:**
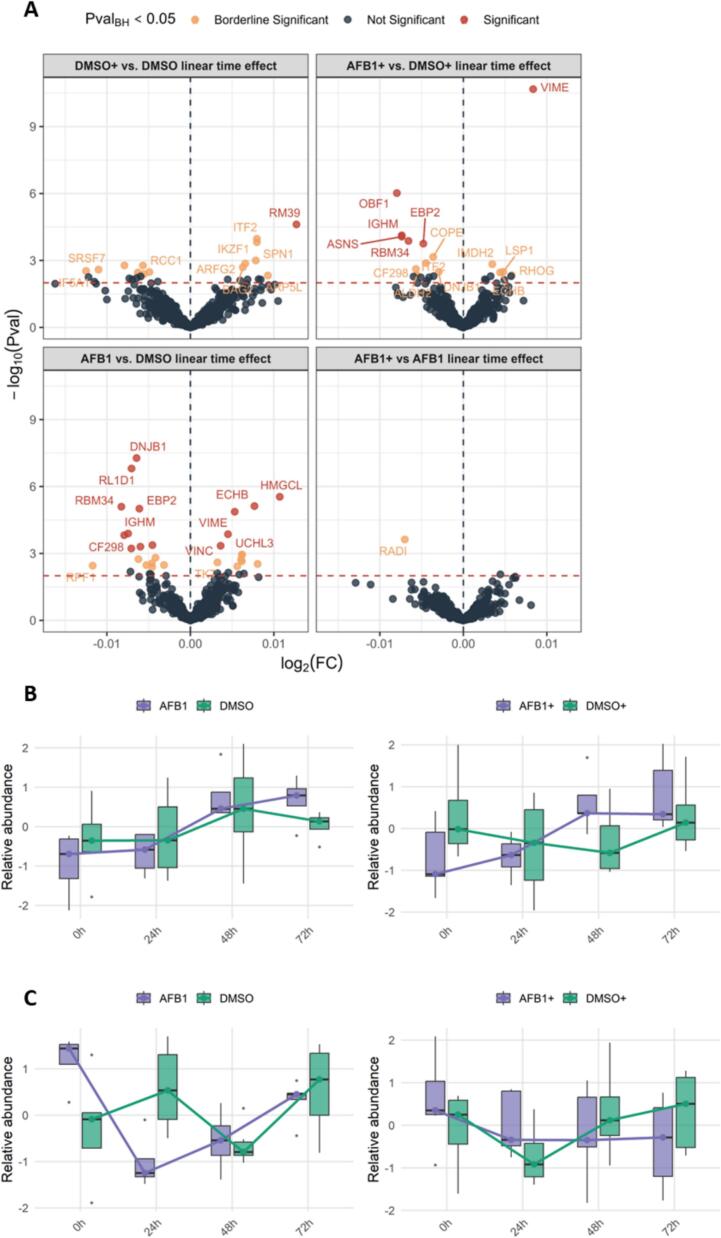
The temporal impact of AFB1 exposure and EBV infection on the acid extractome. (A) Volcano plots illustrating differentially abundant proteins over time for each condition. Significant changes (adjusted p < 0.05) are highlighted in red. Comparisons include DMSO+ vs. DMSO (top left), AFB1+ vs. DMSO+ (top right), AFB1 vs. DMSO (bottom left), and AFB1+ vs. AFB1 (bottom right). (B, C) Box plots depicting temporal changes in VIME (B) and OBF1 (C) protein abundance across 0 h, 24 h, 48 h, and 72 h. AFB1-treated cells compared to DMSO controls in uninfected (AFB1 vs DMSO, left) and EBV-infected (AFB1+ vs DMSO+, right) cells. (For interpretation of the references to color in this figure legend, the reader is referred to the web version of this article.)

Temporal analysis reveals that AFB1 exposure induces only modest changes in protein abundance. Binary log-fold comparisons in abundance at every time point are presented in Supplementary Fig. 4A and further detailed in the interactive report in Supplementary Data 1. However, these snapshots provide only a fragmented view of the cellular response. Therefore, Fig. 4A depicts the differential effects using a linear time model which differs from the conventional binary views (Demeulemeester et al., 2024). Despite the limited number of differentially responding proteins over time, their changes are largely consistent between EBV− and EBV+ cells.

In both infected and uninfected cells, OBF1, RBM34, EBP2 and IGHM change according to a more negative trend following AFB1 exposure compared to DMSO control, while VIME shows a more positive change over time (Fig. 4A). Only one protein is differentially changing in infected cells, i.e. Aspargine Synthetase (ASNS). EBV is associated with 90 % of natural killer T-cell lymphomas (NKTCL), and has been attributed to promoter region hypermethylation, which silences tumour suppressor genes, including that of ASNS, amongst others. Additionally, there are a few more proteins that change in the same direction (DNJB1, RL1D1, CF298, DDX46, RBM28), while a few extra change oppositely (HMGCL, ECHB, UCHL3 and VINC) when uninfected cells are exposed to AFB1 (Fig. 4A). A GO analysis of these significantly negative proteins corrected for the full extractome background again surfaces the pre-ribosome as being significantly enriched (Supplementary Fig. 4D), only here it reaches significance using the significantly changing proteins rather than through GSEA. This is direct evidence that the proteome differences induced by EBV infection at baseline continue to persist or even aggravate during AFB1 incubation. Overall, uninfected cells appear slightly more reactive to AFB1, consistent with their higher translational potential at baseline, a pattern uniquely detectable through the integrated experimental design and statistical approach used in this study.

Fig. 4B and C provide a time-resolved analysis of vimentin (VIME) and POU domain class 2-associating factor 1 (OBF1) abundance trends in infected and uninfected cells, capturing the dynamics illustrated in the linear regression model in Fig. 4A. From this, it is clear that VIME expression is induced by AFB1 between 24 h and 48 h in all cells. For OBF1 downregulation is more pronounced upon AFB1 exposure compared to DMSO in all cells. In the pair-wise comparisons at every time point, this is reflected by the fact that OBF1 is significantly lower in AFB1 treated uninfected cells at 24 h, while it’s only significant at 72 h in infected cells (Fig. 4A; Supplementary Fig. 4A and B). This actually raises the question of whether OBF1 is degraded upon AFB1 infection, rather than reducing its transcription or translation, yet it is impossible to assess this in the current design. Temporal profiling highlights these distinct kinetics: uninfected cells exhibit a rapid decline followed by stabilization, whereas infected cells show a gradual and continuous decrease in OBF1 expression throughout the experiment (Fig. 4B and C). Functionally, OBF1 interacts with POU2F1/OCT1 and POU2F2/OCT2, and is essential for the response of B-cells to antigens. These transcription factors activate the promoters of genes such as histone H2B as well as IL6 and immunoglobulins. This aligns with the observed gradual downregulation of IGHM following AFB1 exposure. Additionally, in herpes simplex virus (HSV) infection, POU2F1 facilitates transcription of viral immediate early genes. Consequently, AFB1-mediated suppression of OBF1 may render Louckes B cells less responsive to viral infections. Conversely, in EBV-infected cells, the delayed downregulation of OBF1 following AFB1 treatment suggests a potential buffering effect of EBV on this regulatory pathway.

These findings suggest that EBV-infected cells exhibit a slower and less pronounced response, consistent with their lower baseline expression of pre-ribosomal proteins at 0 h, indicative of reduced transcriptional and translational activity.

### 3.6 AFB1 induces subtle hPTM changes through time

Binary comparisons at each time point reveal that H3K27me3 increases in all cells (Supplementary Fig. 5A and B). Infected cells appear more epigenetically reactive to AFB1, exhibiting a greater increase in H33K27me3 at the expense of H3K27umod and H3K27ac, particularly at 72 h. While H33K27me3 is also significantly upregulated by AFB1 in uninfected cells, EBV+ cells already showed lower H33K27me3 levels at baseline, potentially allowing for a greater dynamic change over time (Fig. 3). These pairwise analyses also show that H3K9unmod is significantly downregulated after 72 h of AFB1 exposure, while in uninfected cells, the only AFB1-induced PTM significantly differing from DMSO at 72 h is H3S10Ph, a modification linked to transcription and active cell division. Still, direct pairwise comparison of AFB1 treated infected vs uninfected cells is not significantly different within the timeframe of this experiment (Supplementary Fig. 5C and interactive report in Supplementary Data 1).

**Fig. 5:**
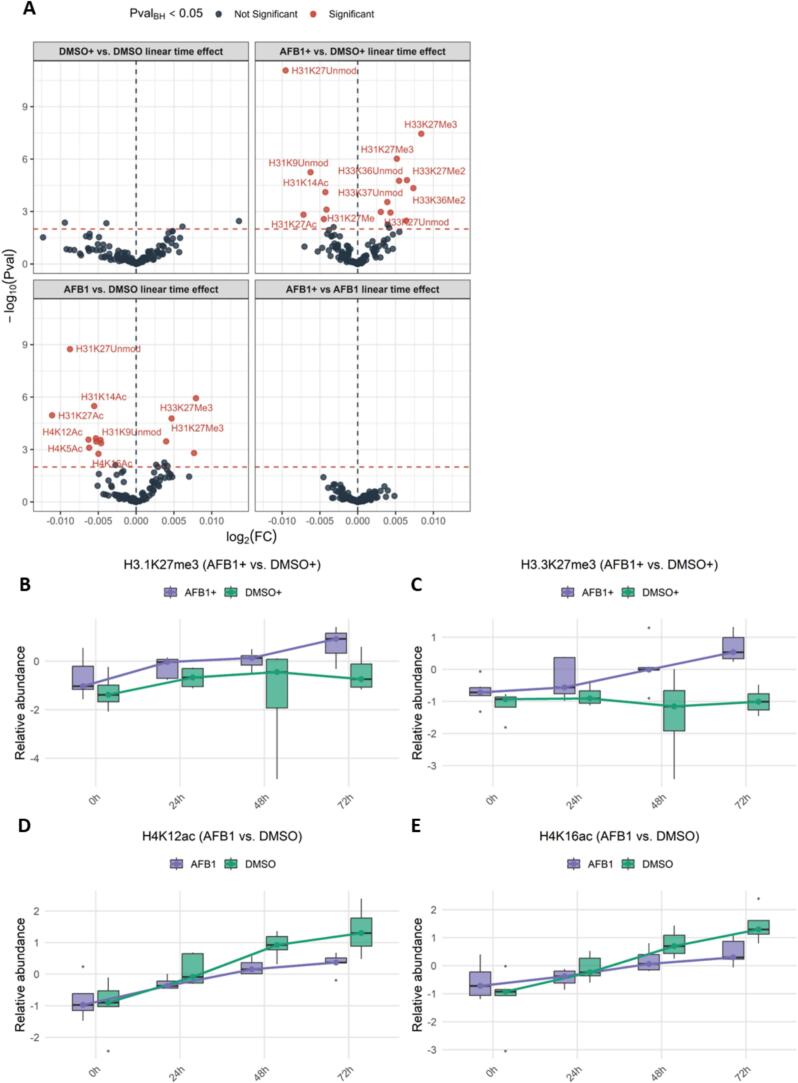
Longitudinal effects of AFB1 exposure and EBV infection on the histone code. (A) Volcano plots showing differentially abundant histone modifications over time. Significant changes (adjusted p < 0.05) are highlighted in red. Comparisons include DMSO+ vs. DMSO (top left), AFB1+ vs. DMSO+ (top right), AFB1 vs. DMSO (bottom left), and AFB1+ vs. AFB1 (bottom right). (B, C) Box plots depicting temporal changes in histone acetylation levels for H3.1K27me3 (B) and H3.3K27me3 (C) in AFB1-treated cells compared to DMSO controls. (D, E) Box plots showing temporal dynamics of H4K12ac (D) and H4K16ac (E) in EBV-infected cells following treatment with AFB1. (For interpretation of the references to color in this figure legend, the reader is referred to the web version of this article.)

Compared to binary comparisons, longitudinal analysis by linear regression modeling captures the changes more directly (Fig. 5A). Indeed, distinct H3K27 dynamics in both infected and uninfected cells are also observed in the longitudinal model, with both H31 and H33 variants being affected (Fig. 5A). An even more detailed inspection of the interactive report in Supplementary Data 1 shows that at least in part, the observed H3K27me3 increase is driven by the incorporation of the trimethylated H33 variant. Variant histone H33 replaces conventional H31 in a wide range of nucleosomes in active genes. It constitutes the predominant form of histone H3 in non-dividing cells and is incorporated into chromatin independently of DNA synthesis. H33 is deposited at sites of nucleosomal displacement throughout transcribed genes, suggesting that it represents an epigenetic imprint of transcriptionally active chromatin.

While these linear regression models reveal significantly altered response over time, they do not directly resolve whether H3K27me3 increases more slowly in AFB1-treated cells or declines more sharply in DMSO-treated cells. A closer examination of H3K27me3 abundance in EBV+ cells over time suggests that the first process contributes most to the observed divergence (Fig. 5B and C). Additionally, these trends are suggestive of a diverging trend that may become more pronounced over extended experimental timeframes. Finally, the incorporation of H33K27me3 in EBV+ Louckes is not affected by DMSO and starts around 24 h, while H31K27me3 diverges less prominently between DMSO and AFB1 and is not apparent until after 48 h.

This approach enables the detection of subtle but distinct hPTM dynamics over time, as illustrated by H4 N-tail acetylation changes that emerge specifically in uninfected cells following AFB1 exposure. Manual inspection illustrates this nicely: H4K12ac and H4K16ac (and H4K5ac, depicted in Supplementary Data 1) clearly raise over time, yet they are induced by DMSO as well. Because of this, binary comparisons at each time point do not show significant changes. However, upon manual inspection, the rise in acetylations in fact goes faster in DMSO than it does for AFB1. In other words, the angle of the linear curve is less steep in AFB1-treated cells, and this causes these PTMs to surpass the significance threshold in the linear regression model, suggesting a meaningful trend (Fig. 5D and E).

In conclusion, AFB1 induces trimethylation of H3K27 on both H31 and H33 at the cost of acetylated and unmodified K27. Thus, while EBV infection induced H3K27ac (at baseline), AFB1 shows the capability of inducing H3K27me3 over (longer) time, therefore displaying an antagonizing effect at this residue. Still both infected and uninfected calls react similarly to AFB1 and this also holds for H3K14ac and H3K9unmod. Only H4 N-tail acetylation seems to be attenuated over time by AFB1 in only uninfected cells, compared to DMSO control.

### 3.7 Does EBV infection disturb the interconnectivity between histone PTMs?

**Fig. 6:**
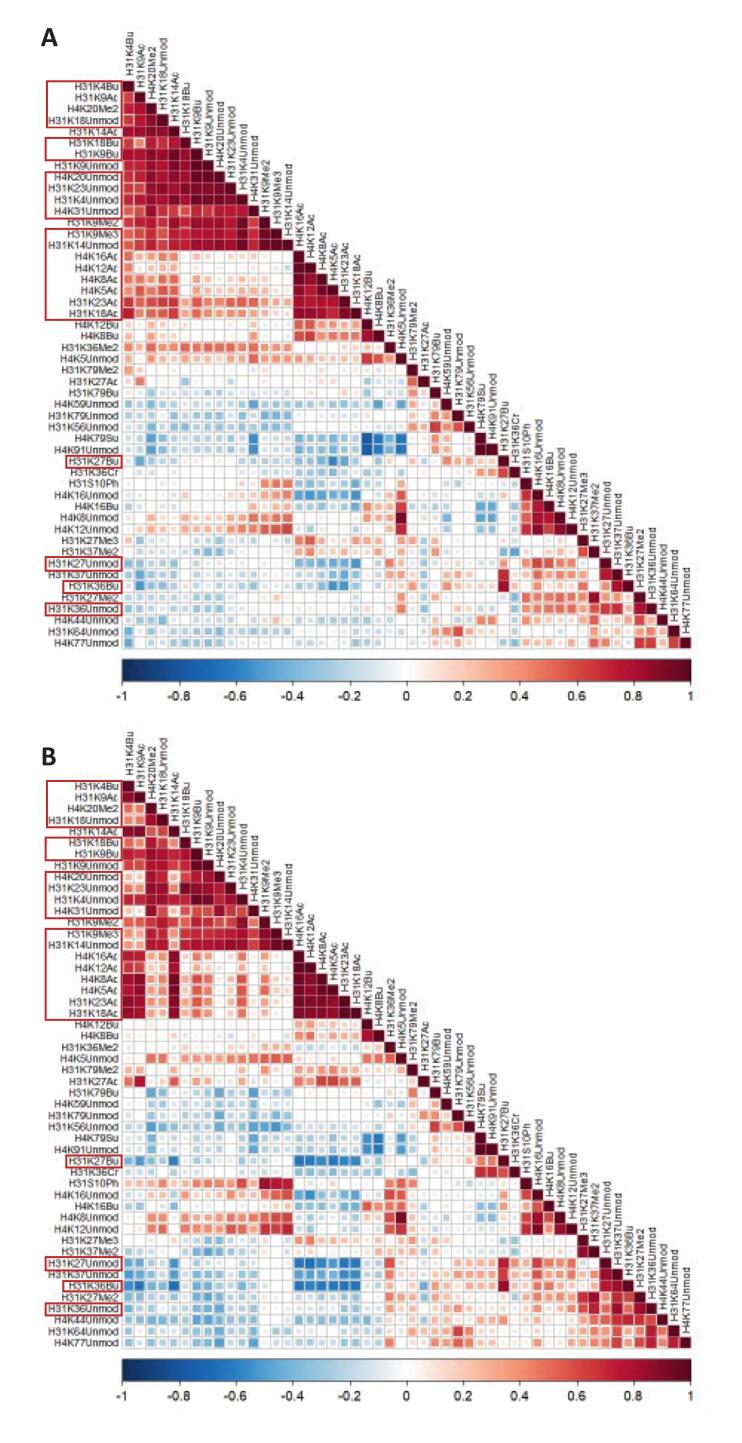
The impact of EBV infection on hPTM covariance. Correlograms showing Pearson correlations between histone post-translational modifications (hPTMs) of H3 and H4 in EBV-negative (A) and EBV-positive (B) Louckes cells exposed to DMSO with or without AFB1 (all timepoints). The analysis quantifies the co-regulation of hPTMs, identifying pairs that consistently change in a similar or opposite manner throughout the experiment. Regions discussed in the main text are highlighted in red. *Note: Butyrylated (bu) residues should be interpreted as methylated (me) sites due to sample preparation, as described in the Methods.* (For interpretation of the references to color in this figure legend, the reader is referred to the web version of this article.)

Beyond changes in single hPTMs, epigenetic regulation operates through interconnected networks, where readers, writers, erasers, and other mediators form dynamic complexes. If EBV infection alters those complexes in any way—such as by displacing a writer protein that binds a specific hPTM—it could disrupt the coordinated response of hPTMs. The combination of untargeted MS with the current time-lapse experimental design provides a unique opportunity to assess these interdependencies by quantifying the co-regulation of hPTMs over time. To explore this, Pearson correlations were computed across all H3 and H4 hPTMs, identifying pairs that always change in a similar or opposite fashion throughout the experiment. As no significant synergistic effects were found for hPTM responsiveness between EBV+ and EBV− cells, we leveraged this approach to specifically study the impact of infection on co-regulation of hPTMs, pooling all DMSO and AFB1 samples to strengthen correlation robustness. Fig. 6A and B show that indeed this approach provides highly reproducible correlograms revealing distinct coordinated patterns between uninfected and infected Louckes cells. In other words, this analysis assesses whether EBV infection alters the coordination of histone modifications rather than their absolute levels, in response to AFB1 and DMSO incubation.

This emergent view of the histone code suggests that EBV infection does not have a drastic impact on the co-regulation of hPTMs in the 72 h timelapse incubation experiment. Still, two patterns are shifting slightly in EBV-infected cells. On the one hand, co-regulation reduces between [H31(K4bu + K9ac + K18ac)], [H4(K20unmod/me2 + K31unmod) and H31(K9bu/me3 + K14unmod + K18unmod/bu + K23unmod)]. On the other hand, acetylated [H4(K5ac + K8ac + K12ac + K16ac) N-tail as well as H31(K18ac + K23ac)] cluster intensifies its co-regulation with [H31(K4bu + K9ac + K18ac)] and intensifies its anti-correlation with [H31(K27unmod/bu + K36 unmod/bu].

**Fig. 7:**
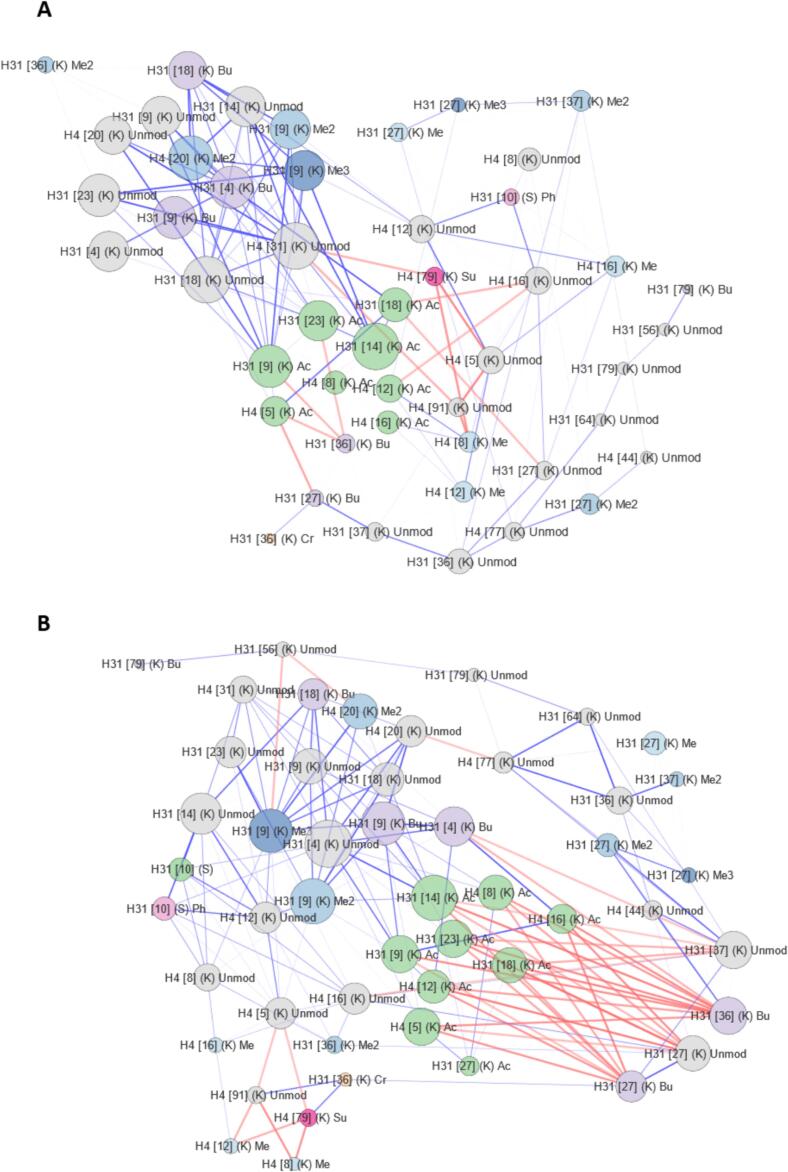
Network plots showing hPTM-hPTM covariance over all samples in the design for EBV-negative (A) and EBV-positive (B) Louckes cells. Nodes represent individual histone modifications, with node size indicating correlation degree. Edges represent Pearson correlation coefficients with negative correlations shown in blue and positive correlations shown in red. Edge transparency corresponds to absolute correlation coefficient. *Note: Butyrylated (bu) residues should be interpreted as methylated (me) sites due to sample preparation, as described in the Methods.* (For interpretation of the references to color in this figure legend, the reader is referred to the web version of this article.)

To better visualize these subtle alterations, correlation matrices were translated into hPTM networks at a 0.5 threshold, highlighting interconnected hubs within the histone modification landscape (Fig. 7A and B). The intensity of the correlation is represented by edge width, while edge color indicates whether the correlation is positive or negative. These hPTM networks better reveal how EBV infection changes the connectivity of the histone PTM landscape in response to AFB1 and DMSO exposure. Now, it becomes even more clear that H3K27/36unmod and H3K27/36bu become integrated into the more tightly clustered H3/H4 acetylation hub formed in infected cells and that unmodified and monomethylated H3K4 and H3K9 anti-correlate this acetylation hub to the other PTMs. In other words, all acetylations on the H4 N-tail (K5, K8, K12, K16) and H3 N-tail (K9, K14, K23, K27) move in a more coordinated manner in EBV+ cells and do so in positive correlation with unmodified and monomethylated H3K27/K36, and in anti-correlation with unmodified and monomethylated H3K4 and H3K9.

## 4 Discussion

In this study, we investigated the impact of EBV infection and AFB1 exposure on the histone code and a subset of the proteome. A simple binary comparison between AFB1 0 h and AFB1 72 h, reveals a widespread upregulation of histone PTMs, particularly acetylations (Supplementary Fig. 6). However, while DMSO is commonly used as a negative solvent control, we find that acetylations also significantly increase within 72 h in this negative control, thereby reducing analytical depth. Alternatively, independent of DMSO, these acetylations could reflect the epigenetic manifestation of an exponential growth phase. If the increasing acetylations on the H4 N-tail are induced by the solvent alone, this will have a downstream impact on gene activation in ways that do not reflect natural AFB1 exposure. Still, we could detect that in uninfected cells, AFB1 attenuates this DMSO-induced acetylation underscoring the potential of a careful experimental design and statistical rigor. Our timelapse approach represents one of the few studies to track hPTM dynamics over time (De Clerck et al., 2019) and to our knowledge, is the first to assess AFB1-induced alterations in hPTMs and co-extracted proteins in BL cells. Therefore, these findings provide the necessary step before more mechanistically oriented follow-up experiments are considered.

Irrespectively, hPTMs require only subtle changes in a bulk analysis to attain potentially large phenotypic impacts, such as the loss of a modification at a promoter region regulating a key cellular mediator protein. In this untargeted hPTM analysis, all histones were extracted from the whole genome and compared to other omics studies, more subtle shifts need to be detected when studying histonomics. This level of resolution was achievable through a tightly controlled experimental design with large sample numbers. However, increasing the scale of the experiment inevitably amplifies the risk of batch effects. Together with the confounding effect of DMSO this underscored the need for more advanced statistical processing, which was enabled by the modelling framework proposed by msqrob2PTM and the timelapse design of the experiment. This approach enabled extensive normalization at multiple levels, inferred the abundance of all modified and unmodified lysine residues from the measured abundance of the peptidoforms, and to construct both factor and linear time regression models. These models not only allowed the assessment of differential hPTM abundance at individual time points but also captured dynamic changes over time. The robustness of this method is evident in the consistency of findings between independent comparisons of uninfected and infected cells. Inversely, this consistency also suggests that EBV infection is not profoundly changing the effects that are induced by AFB1 (and DMSO).

From the perspective of EBV infection, we observed a reduction in a few key proteins involved in transcription and translation, alongside the downregulation of immunological mediators. Overall, this makes the uninfected cells more primed to phenotypic reaction upon challenge. At the histone level however, infection is reflected by a reduced H3K27me3, increasing the amount of acetylated and unmodified H3K27 in the process. The only differentially used histone variant is H33 in infected cells (Supplementary Data 1). Interestingly, EBV maintains infection latency through H33 incorporation a process regulated by H3 modifications such as methylations at H3K9 and H3K27 (Zhang et al., 2020). Additionally, EBV induces the expression of KDM6B; a histone demethylase that removes me3 at H3K27 contributing to lymphoma development (Anderton et al., 2011). These mechanisms likely explain the observed decrease in H3K27me3. Taken together, EBV-infected Louckes exhibit reduced protein synthesis capacity, while increased acetylation potentially enhances chromatin accessibility.

From the perspective of AFB1 exposure, regardless of infection status, four proteins—OBF1, RBM34, IGHM, and EBP2—decline more readily in response to AFB1 compared to the DMSO control. Whether this reduction results from decreased expression and translation or increased protein degradation remains unclear, though the time-course data suggests the latter. These proteins are associated with key B-cell functions, including germinal center formation (OBF1), lymph node activity (RBM34), IgM production (IGHM), and ribosomal large subunit biogenesis (EBP2). Notably, VIME is upregulated, which plays a key role in B-cell activation by facilitating receptor signaling, antigen internalization, intracellular trafficking, and antigen presentation (Tsui et al., 2018). AFB1-induced VIME upregulation may represent a compensatory response to immune dysregulation. Together, these findings suggest that AFB1 alters the expression and/or degradation of several immunologically relevant proteins irrespective of EBV infection. Still, several more proteins were differentially affected in uninfected cells that are strongly involved in the pre-ribosome machinery, reinforcing the idea that translation remains more intact in uninfected cells compared to infected ones and contributes significantly to the observed proteomic changes.

Overall, we have no direct evidence for synergistic or antagonistic interactions of EBV infection and AFB1 exposure. However, Pearson correlations between all hPTMs over time, combining both AFB1 and DMSO incubations, reveal that infected cells display a more coordinated behavior of acetylation on the H3 and H4 N-tails compared to uninfected cells. This observation is in line with the role of EBV-encoded proteins interacting with histone acetyltransferases and methyltransferases, facilitating chromatin remodeling to regulate genes involved in cell proliferation, angiogenesis, and immune evasion (Scott, 2017, Tempera and Lieberman, 2014). Our findings further reveal that the acetylation cluster of PTMs correlates more strongly with monomethylated H3K27 and H3K36, while anticorrelating with unmodified and monomethylated H3K4 and H3K9. It is known that the chromatin state required for EBV lytic gene activation involves transcription activation markers, such as H3K27ac, H3K9ac, and H4K8ac, and repressive markers, such as H3K27me3, H3K9me2/me3, and H4K20me3, yet it is not straightforward to interpret these earlier findings in the context of our results (Countryman et al., 2008, Ramasubramanyan et al., 2012). Irrespectively, our findings suggest that AFB1 exposure alters the coordination of these marks, potentially modulating the kinetics or stability of EBV chromatin remodeling. Furthermore, EBV-mediated epigenetic remodeling extends to broader oncogenic signaling, immune regulation, and metabolic reprogramming (Bauer et al., 2021). Acetylation is intrinsically linked to metabolism and promotes chromatin accessibility, facilitating immune evasion—an essential feature of EBV persistence and pathogenesis (Sun et al., 2022). Our findings reveal that in EBV+ cells, histone acetylation becomes more coordinated, integrating H3K27 into a broader acetylation hub, which may reflect a chromatin state favoring transcriptional activation and viral persistence. Given that EBV drives glutamine addiction and methionine depletion, both of which regulate histone methylation and viral latency (Guo et al., 2022, Pan et al., 2016, Xiong et al., 2021) this reorganization may reflect epigenetic adaptation to metabolic stress, linking histone PTM connectivity to EBV-driven oncogenesis.

Considering that epigenetic regulation is a major driver of EBV pathogenesis, targeting epigenetic modifications is a promising strategy in treating EBV-associated diseases. The histone deacetylase inhibitor (HDACi) vorinostat shows favorable anti-tumor activity *in vitro* in lymphoma cells (Buglio et al., 2008). The DNA methyltransferase inhibitor (DNMTi) decitabine regulates the EBV latency patterns in BL cells, enhancing sensitivity to EBV-specific cytotoxic T lymphocytes in preclinical studies (Dalton et al., 2020). Increasing clinical trials are evaluating HDACis and DNMTis as both monotherapies and in combination for treating EBV-associated diseases (Rosenthal et al., 2023, Sborov et al., 2017). Among HDACis, mocetinostat and entinostat demonstrated disease control rates of 35 % and 24 %, respectively, in Phase 2 trials enrolling relapsed lymphoma patients (Batlevi et al., 2016, Younes et al., 2011). However, challenges still remain such as, the reported rodimepsin-induced EBV reactivation in patients with natural killer/T-cell lymphoma, highlighting the complexity of targeting epigenetic regulators in EBV-driven malignancies (Kim et al., 2016).

Despite these insights, our findings stem from differential co-regulation analysis rather than differential abundance, making interpretation challenging. This warrants further investigation to delineate the mechanistic implications.

### 4.1 Study limitations

This study has several limitations that should be considered when interpreting the findings. First, a suited negative control model turns out to be very challenging. Although DMSO is commonly used as a vehicle control, our results show that DMSO displayed significant increases in histone acetylation over time, particularly on the H4 N-terminal tail. While this could be independent from DMSO itself and rather indicate an exponential growth phase in the cell culture, this is the only vehicle control available. Therefore, these DMSO-induced changes partially reduce analytical contrast and may confound interpretation of treatment effects. Still, we demonstrate that adequately complex experimental design and statistical rigor can isolate the differences with the negative control, allowing us to conclude that AFB1 attenuated some of these acetylation effects in uninfected cells.

Second, the study relied on untargeted hPTM profiling using histones extracted from the entire genome. Compared to gene- or locus-specific omics approaches like CUT&TAG, this requires detection of subtle shifts that may still carry strong phenotypic consequences, such as altered promoter regulation of key transcriptional mediators. Detecting these changes demands large sample sizes and high-resolution analysis. However, scaling up the experiment also increases susceptibility to batch effects, which were addressed here using a robust statistical framework (msqrob2PTM) and a time-lapse design allowing for multi-level normalization and dynamic modeling. Also, limited sample numbers intrinsically require choices, e.g. the time span that is investigated. In this study, we therefore only started to pick up the divergences in H3K27me3 at 72 h, yet a projection based on these trends predicts that this difference will only expand after longer-term exposures. A comprehensive and detailed results report is therefore provided for the readership to manually inspect and search for specific targets of interest (Supplementary Data 1).

Third, while we observed a consistent decline in proteins such as OBF1, RBM34, IGHM, and EBP2 following AFB1 exposure, the current design does not distinguish between reduced transcription, impaired translation, or increased degradation. Further mechanistic work including transcriptomic and targeted degradation assays will be necessary to clarify the pathways involved.

Finally, while correlation-based methods capture dynamic relationships between modifications, these findings are very hard to confirm in mechanistic follow-up experiments as they are indirect indications that regulatory protein complexes are differentially assembled or display reduced activity.

## 5 Conclusions

This study provides new insights into the interplay between AFB1 exposure and EBV infection in shaping the histone modification landscape of BL cells. Our findings highlight the dynamic changes in hPTM changes induced by AFB1 (and DMSO) over time including methylation status of H3K27 and to some extent acetylation of H4 N-tail. The quantitative changes of single hPTMs induced by AFB1 are subtle and are not different between infected and uninfected cells. Yet, our time-resolved analysis revealed subtle but significant temporal trends in hPTMs, particularly in acetylation patterns of H4 N-tail residues. The observed differences in protein expression and hPTM dynamics suggest that AFB1 exposure affects key cellular processes related to B-cell function, potentially impairing immunological responsiveness. The integration of correlation and network-based approaches provides deeper insights into the coordinated regulation of hPTMs, revealing subtle alterations in co-regulation of H4 and H3 N-tail acetylations and unmodified/monomethylated H3K27/K36 between infected and non-infected cells.

## 6 Disclaimer

“Where authors are identified as personnel of the International Agency for Research on Cancer/World Health Organization, the authors alone are responsible for the views expressed in this article and they do not necessarily represent the decisions, policy or views of the International Agency for Research on Cancer/World Health Organization.”

## CRediT authorship contribution statement

**Thanos M. Michailidis:** Conceptualization, Data curation, Formal analysis, Investigation, Methodology, Project administration, Validation, Visualization, Writing – original draft. **Laura Corveleyn:** Writing – original draft, Visualization, Project administration, Methodology, Investigation, Formal analysis, Data curation, Conceptualization. **Ruben Almey:** Writing – original draft, Investigation, Data curation. **Yasmine Bader:** Writing – review & editing, Investigation. **Grace A. Odongo:** Writing – review & editing, Investigation. **Zdenko Herceg:** Writing – review & editing. **Rita Khoueiry:** Writing – review & editing, Supervision, Investigation. **Sarah De Saeger:** Writing – review & editing, Supervision. **Marthe De Boevre:** Writing – review & editing, Supervision, Project administration, Conceptualization. **Maarten Dhaenens:** Writing – review & editing, Supervision, Project administration, Conceptualization.

## Funding

This work was financially supported by the FWO grant G085921N and the FWO mandate 1SF2624N. TMM is funded as a predoctoral researcher under the G085921N program and LC is funded as a predoctoral researcher under 1SF2624N. RA is funded by the Ghent University Special Research Fund (BOF21/DOC/076). MDB has received funding from the European Research Council (ERC) under the European Union’s Horizon 2020 research and innovation program (grant agreement No 946192, HUMYCO).

## Declaration of competing interest

The authors declare that they have no known competing financial interests or personal relationships that could have appeared to influence the work reported in this paper.

## Data Availability

The mass spectrometry proteomics data along with Supplementary Data 1 have been deposited to the ProteomeXchange Consortium via the PRIDE (Perez-Riverol et al., 2025) partner repository with the dataset identifier PXD060719 and 10.6019/PXD060719. For the reviewing process only they can be accessed with the credentials found in Supplementary Data 2.
