# Supplementary material for: Aflatoxin B1 induces subtle but coordinated histone modifications in Epstein-Barr virus infected and non-infected Burkitt lymphoma cells
Source: Environ Int. 2025 Oct;204:109813. doi: 10.1016/j.envint.2025.109813 (PMC12715419; doi:10.1016/j.envint.2025.109813)
Supplement: Supplementary Data 1 [file mmc1.zip › mmc1.html]

Differential hPTM usage


# Differential hPTM usage

 Code

- Show All Code
- Hide All Code
- ---
- View Source

EpiMycoTox — Thanos Mouchtaris-Michailidis

Author

Affiliation

Ruben Almey

Progentomics, Ghent University

Published

December 10, 2024

## Table of contents

- 1 Overview
- 2 Data preprocessing
  - 2.1 Data import
  - 2.2 Sample QC
  - 2.3 Filtering
  - 2.4 Normalization
- 3 Differential usage analysis
  - 3.1 Model building
  - 3.2 Statistical testing

## 1 Overview

This workflow is an adaptation of msqrob2PTM1. In brief, data is imported, filtered, and normalized in two steps (general and usage normalization). A peptide ion data export from Progenesis QIP2 is preprocessed in Section 2 and differential abundance analysis is then performed in Section 3.

We start by importing the relevant packages.

Code

```
# tidyverse
library(tidyverse)
# proteomics
library(QFeatures)
library(msqrob2)
# custom functions
source("./src/consensus_hPTM_utils.R")
```

## 2 Data preprocessing

### 2.1 Data import

First, read in the hPTM dataset (all peptide ion export from Progenesis QIP) and transform into a QFeatures3 object. This data contains all identified peptide ions with their lfq abundances per run, histone features being feature edited and tagged in Progenesis QIP.

Code

```
# read the histone dataset
quantcol_pattern <- "231030_"
df_histone <- read_csv("./data/processed/241202_peptide_export_all_proteins_parsed.csv", col_types = cols(
    feat = col_integer(),
    protein = col_character(),
    histone = col_logical(),
    contaminant = col_logical(),
    sequence = col_character(),
    charge = col_integer(),
    mods = col_character(),
    .default = col_double()
)) %>%
    mutate(
        precursor = paste(sequence, charge, mods, sep = "_"),
        protein = str_replace_all(protein, ";", "/"),
        family = str_extract(protein, "^H(1|2A|2B|3|4)"),
        core = grepl("^H(2A|2B|3|4)", family)
    )
# read metadata
colData <- read_csv("./data/raw/epimycotox_thanos_metadata.csv", col_types = cols(
    Sample_name = col_character(),
    Include = col_logical(),
    Outlier = col_logical(),
    Treatment = col_factor(),
    EBV = col_factor(),
    Time = col_integer(),
    Replicate = col_factor(),
    Extraction_batch = col_factor()
))
# check for mismatch between samples in quantitative dataset and metadata
common_samples <- intersect(colnames(df_histone), colData$Sample_name)
quant_not_meta <- setdiff(grep(quantcol_pattern, colnames(df_histone), value = TRUE), colData$Sample_name)
if (length(quant_not_meta) > 0) {
    stop(sprintf(
        "Samples in quantitative data but not in metadata, add to metadata .csv: %s",
        paste(quant_not_meta, collapse = ", ")
    ))
}
meta_not_quant <- setdiff(colData$Sample_name, colnames(df_histone))
if (length(meta_not_quant) > 0) {
    dropped <- paste(meta_not_quant, collapse = ", ")
    colData <- colData[colData$Sample_name %in% common_samples, ]
}
# simplify sample names and specify groups
df_histone <- rename_with(
    df_histone,
    str_replace_all,
    pattern = paste0(quantcol_pattern, "|_02"),
    replacement = ""
)
colData <- arrange(colData, match(common_samples, Sample_name)) %>%
    mutate(quantCols = str_replace_all(
        Sample_name,
        pattern = paste0(quantcol_pattern, "|_02"),
        replacement = ""
    ), .keep = "unused") %>%
    mutate(
        Group = paste(Treatment, EBV, Time, sep = "_"),
        Treat_EBV = paste(Treatment, EBV, sep = "_"),
        across(c(Group, Treat_EBV), as.factor)
    )
# to QFeatures object
pe <- readQFeatures(
    df_histone,
    colData = colData,
    name = "precursorRaw",
    verbose = FALSE
)
if (length(meta_not_quant) > 0) {
    print(sprintf(
        "Samples in metadata but not in quantitative data, dropping: %s",
        str_replace_all(dropped, pattern = quantcol_pattern, replacement = "")
    ))
}
```

Inspect if the following looks correct:

- Precursor metadata
- Sample metadata

### 2.2 Sample QC

Next, we can inspect the peptide loading consistency and histone extraction efficiency.

Code

```
# sample loading
nonhistone_assay <- assay(pe[!rowData(pe[["precursorRaw"]])$histone, ], "precursorRaw")
median_raw_nonhistone_abundance <- colMedians(log2(nonhistone_assay), na.rm = TRUE)
colData(pe)[names(median_raw_nonhistone_abundance), "median_raw_nonhistone_abundance"] <- median_raw_nonhistone_abundance
# histone loading
histone_assay <- assay(pe[rowData(pe[["precursorRaw"]])$histone, ], "precursorRaw")
median_raw_histone_abundance <- colMedians(log2(histone_assay), na.rm = TRUE)
colData(pe)[names(median_raw_histone_abundance), "median_raw_histone_abundance"] <- median_raw_histone_abundance
# visualize loading consistency / histone extraction efficiency
p <- ggplot(
    colData(pe[, !grepl("QC_", pe$quantCols)]),
    aes(x = median_raw_histone_abundance, y = median_raw_nonhistone_abundance, color = Extraction_batch, shape = Outlier)
) +
    geom_point() +
    geom_smooth(aes(color = NULL, shape = NULL), se = FALSE) +
    geom_abline(intercept = 0, slope = 1)
p <- ggExtra::ggMarginal(p, groupFill = TRUE, position = "stack")
grid::grid.newpage()
grid::grid.draw(p)
```

Figure 1: Evaluation of histone loading per extraction batch

Clear differences in extraction efficiency can be seen between extraction batches. This will need to be corrected for during modelling. Some outlier samples will be removed although nothing extreme.

### 2.3 Filtering

Remove precursors with less than 75 % presence.

Code

```
# drop outliers
pe <- pe[, !pe$Outlier]
rows_before_filter <- nrow(pe[["precursorRaw"]])
# drop contaminant features
pe <- filterFeatures(pe, ~ !contaminant)
# remove high missing features
pe <- zeroIsNA(pe, i = "precursorRaw")
pe_nNA <- nNA(pe, i = "precursorRaw")
# at least 3 samples should have the precursor quantified + 75 % across
pe <- pe[!(ncols(pe)[[1]] - pe_nNA$nNArows$nNA < 3 | pe_nNA$nNArows$pNA > 0.25), ] %>%
    # simplify protein grouping, for co-extracts?
    filterFeatures(~ protein %in% smallestUniqueGroups(protein, split = "/"))
```

This leaves us with 4623 observations (516 rows dropped). We can now look at the histone family coverage.

Code

```
# plot remaining
rowData(pe[rowData(pe[["precursorRaw"]])$histone, ][["precursorRaw"]]) %>%
    ggplot(aes(
        x = family,
        fill = !is.na(mods) & !sapply(str_split(mods, ";"), function(x) all(grepl("Unmod", x)))
    )) +
    geom_bar() +
    labs(fill = "Modified")
```

Figure 2: Number of unique precursors (peptide + charge + modifications if any) quantified per histone family

### 2.4 Normalization

Normalization of the samples is performed in two steps:

1. Removal of technical variation, such as from sample loading differences, using variance stabilization (VSN)4. This normalizes all samples based on the abundance distribution of all precursors
2. Correction of histone variant/nucleosome abundance differences to allow differential **usage** analysis of histone variants and PTMs.

#### 2.4.1 Global normalization

Start with VSN normalization and evaluate the results.

Code

```
# VSN requires the non-log transformed values as it glog transforms the data
pe <- normalize(pe, i = "precursorRaw", method = "vsn", name = "precursor", verbose = FALSE)
# normalized histone abundances
histone_assay <- assay(pe, "precursor")
median_histone_abundance <- colMedians(histone_assay, na.rm = TRUE)
colData(pe)[names(median_histone_abundance), "median_histone_abundance"] <- median_histone_abundance

# visualize
vsn::meanSdPlot(assay(pe[["precursor"]]))
d <- limma::plotDensities(assay(pe[["precursor"]]), col = as.numeric(pe$Group), legend = FALSE)
boxplot(assay(pe[["precursor"]]), col = pe$Group, ylab = "Intensity")
```

(a) MeanSdPlot for quality check of VSN normalization: the red line should be approximately horizontal

(b) Density plot of normalized precursor abundances per sample, coloured by extraction batch

(c) Boxplot of normalized precursor abundances per sample, coloured by extraction batch

Figure 3: Evaluation of global normalization

One sample (DMSO\_72h\_6) looks different from the rest. This sample will also be discarded.

Code

```
pe <- pe[, -which.max(apply(d$Y, 2, max))]
limma::plotDensities(assay(pe[["precursor"]]), col = as.numeric(pe$Group), legend = FALSE)
```

Figure 4: Evaluation of global normalization after outlier removal

Peptide-level MDS plots, color-coded according to the metadata, will show the level of similarity between samples. Look for any obvious clustering, if present. Do note that this is based on all peptide data, including from co-extracts.

- Include
- Treatment
- EBV
- Time
- Replicate
- Extraction\_batch
- median\_raw\_nonhistone\_abundance
- median\_raw\_histone\_abundance
- median\_histone\_abundance

From the MDS plots, we can see that the extraction batch (clear clusters) and loading differences (median histone abundance ~ PC2) are the main drivers of dissimilarity between samples, even after normalization. We will need to take this into account during the modelling step.

We can already aggregate the co-extracts to the protein level for later testing.

Code

```
# select co-extracts and do a second mode between protein fold change normalization, as per MS-DAP
pe <- addAssay(pe, subset(pe[["precursor"]], subset = !histone), "precursorCoextr") %>%
    addAssayLink("precursor", "precursorCoextr", "feat", "feat") %>%
    normalize_usage(
        i = "precursorCoextr",
        fcol = "protein",
        name = "precursorCoextrMB",
        groups = "Group",
        scaling_fun = "FCBM",
        aggregation_fun = MsCoreUtils::robustSummary
    )
# aggregate co-extracts
pe <- aggregateFeatures(
    pe,
    i = "precursorCoextrMB",
    fcol = "protein",
    name = "co-extracts",
    fun = MsCoreUtils::robustSummary
)
```

#### 2.4.2 Usage normalization

To perform the second normalization step, we first create a histone assay in which we deconvolute the data to the single PTM level.

Code

```
# deconvolute histone peptidoforms based on sPTM definition
pe <- deconvolute(
    pe,
    i = "precursor",
    fcol = "precursor",
    deconv = "mods",
    top_level = "protein",
    name = "precursorDeconv",
    filter = ~histone
)
```

A total of 146 single PTMs were defined. Now we perform the second normalization step. We do this by centering the fold change differences between sample groups at both the histone variant and PTM level, i.e., fold change distributions for histone variant and PTM abundance between two sample groups should center around zero.

Code

```
# normalize histone peptidoforms
pe <- normalize_usage(
    pe,
    i = c("precursorMods", "precursorDeconv"),
    fcol = list("precursorMods" = "family", "precursorDeconv" = "deconvoluted"),
    name = c("precursorMB", "precursorDeconvMB"),
    combine_fcol = TRUE,
    groups = NULL,
    sep_level = "core",
    scaling_fun = "FCBM",
    aggregation_fun = MsCoreUtils::robustSummary
)
```

Now aggregate to the single PTM level, starting from the relevant assays, and perform some QC checks.

Code

```
# helper function
as_matrix <- function(x) {
    y <- as.matrix.data.frame(x[, -1])
    rownames(y) <- x[[1]]
    y
}

logfc_means <- list()
shapiro_test <- list()
# every entry of the list will be one assay to aggregate and check between-group logFC modes
for (viz_assay in list(
    c("precursorMods", "protein", "variant_noMB"),
    c("precursorMB", "protein", "variantMB"),
    c("precursorDeconv", "deconvoluted", "ptm_noMB"),
    c("precursorDeconvMB", "deconvoluted", "ptmMB")
)) {
    # calculate pairwise logFC from assay
    pe <- aggregateFeatures(
        pe,
        i = viz_assay[[1]],
        fcol = viz_assay[[2]],
        name = viz_assay[[3]],
        fun = MsCoreUtils::robustSummary, # consider medianPolish if this is too slow, scales better
        na.rm = TRUE
    )
    # calculate between-group logfc modes
    logfc_modes <- assay(pe, viz_assay[[3]]) %>%
        t() %>%
        as_tibble() %>%
        group_by(pe$Group) %>%
        summarise(across(everything(), ~ mean(.x, na.rm = TRUE))) %>%
        as_matrix() %>%
        t() %>%
        pairwise_modes()
    # rowmedians and normality test for every pairwise logfc comparison
    logfc_means[[viz_assay[[3]]]] <- rowMeans(logfc_modes)
    shapiro_test[[viz_assay[[3]]]] <- apply(logfc_modes, 1, shapiro.test)
    # density plot
    limma::plotDensities(logfc_modes,
        legend = FALSE, col = "darkgray",
        main = paste("Pairwise between-group LogFC, assay", viz_assay[[3]])
    )
    # add overall mean density to plot
    d <- apply(logfc_modes, 1, function(x) {
        density(x,
            bw = "SJ", from = range(logfc_modes)[[1]],
            to = range(logfc_modes)[[2]], na.rm = TRUE
        )
    })
    matlines(d[[1]]$x, rowMeans(sapply(d, function(x) x$y)), lwd = 6, col = "darkviolet")
    abline(v = 0, col = "gray20", lty = "dashed", lwd = 2)
}
```

(a) Protein-level, before usage normalization

(b) Protein-level, before usage normalization

(c) PTM-level, before usage normalization

(d) PTM-level, after usage normalization

Figure 5: Distribution of pairwise logFC modes between sample groups (gray) and their overall mean distribution (purple).

LogFC differences between sample groups center well around 0, more so at the PTM level than at the histone variant level (more ambiguity, so not unexpected). Usage normalization decreased the spread of these distributions, correcting for “nucleosome abundance” differences between samples.

Code

```
as_tibble(logfc_means[c("variant_noMB", "ptm_noMB")]) %>%
    mutate(row_mean = abs(rowMeans(across(everything())))) %>%
    mutate(across(everything(), ~ signif(., 4))) %>%
    mutate(group = names(logfc_means[[1]]), .before = 1) %>%
    arrange(desc(row_mean))
lapply(shapiro_test[c("variantMB", "ptmMB")], function(x) {
    sapply(x, function(y) y$p)
}) %>%
    as_tibble() %>%
    mutate(row_mean = rowMeans(across(everything()))) %>%
    mutate(across(everything(), ~ signif(., 4))) %>%
    mutate(group = names(shapiro_test[[1]]), .before = 1) %>%
    arrange(row_mean)
```

Table 1: Summary statistics of pairwise logFC mode between sample calculations at both the histone variant and PTM level.

(a) Absolute mean logFC modes between sample groups before usage normalization, sorted in decreasing order. Higher values signify large differences compared to other sample groups, technical (e.g. low histone extraction yield) or biological (e.g. high cell death, …).

(b) Shapiro-Wilk p values of pairwise logFC mode distributions between sample groups after usage normalization, sorted by increasing mean. Low p values mean more evidence against normality; sample groups with low p values may be highly differential/outliers.

Now we can repeat the MDS analysis, but instead of the globally normalized peptide-level data we now only use histone data at different assay levels:

- Left, usage normalized **precursor** level
- Middle, usage normalized **single PTM** level through robust summarization from deconvoluted precursor level
- Right, usage normalized **histone variant** level through robust summarization from precursor level

MDS plots of all samples after general and usage normalization:

- Include
- Treatment
- EBV
- Time
- Replicate
- Extraction\_batch
- Treat\_EBV
- median\_raw\_nonhistone\_abundance
- median\_raw\_histone\_abundance
- median\_histone\_abundance

The extraction batch remains the main driver of dissimilarity between samples. No other clusters are apparent. Finally, the preprocessed dataset and all its assays can be represented as such:

Figure 6: Visual representation of the QFeatures object holding all data and its relations

## 3 Differential usage analysis

### 3.1 Model building

We will start by creating the model formula and relevant contrasts, then we can proceed with differential usage testing. First, how should we model the time effect?

- variantMB
- precursorMB
- ptmMB

As there is only one timepoint of VPA, we cannot include these samples in the timeseries design. We will make three models: one “means model” with time as a factor, one “regression model” with linear time as a covariate, and one regression model with quadratic time as a covariate.

Code

```
# drop runs not in the "Include" column of colData, e.g., QC runs or outliers
# also drop VPA samples, as we only have 1 timepoint for these
pe <- pe[, pe$Include & !pe$Treatment == "VPA"]
colData(pe) <- droplevels(colData(pe))
# relevel factors as needed
pe$Treatment <- relevel(pe$Treatment, "DMSO")
pe$Treat_EBV <- relevel(pe$Treat_EBV, "DMSO_Neg")
# establish the correct formula and explore the design, do not yet include random effects
# ExploreModelMatrix::ExploreModelMatrix(colData(pe)[, !apply(colData(pe), 2, function(x) any(is.na(x)))])
formulas <- list()
formulas[["factor"]] <- "~ 0 + Treat_EBV * as.factor(Time)"
formulas[["timeseries_linear"]] <- "~ 0 + EBV + Treat_EBV:Time"
formulas[["timeseries_quadratic"]] <- "~ 0 + EBV + Treat_EBV:poly(Time, degree=2)"
model_designs <- list()
for (designFormula in formulas) {
    model_designs[[designFormula]] <- ExploreModelMatrix::VisualizeDesign(
        colData(pe),
        designFormula = as.formula(designFormula),
        textSizeFitted = 2
    )
    if (designFormula == "~ 0 + Treat_EBV * as.factor(Time)") { # only really informative for factor model
        purrr::walk(model_designs[[designFormula]]$plotlist, print)
    }
}
```

Figure 7: Visualization of the factor design matrix.

We can include other model parameters to account for random variation: typical examples are loading differences or sample preparation batches. For random effects, we can estimate the intra-block correlation to determine whether or not to include these as random effects.

Code

```
# check if we should include random effects
for (i in seq_along(formulas)) {
    limma::duplicateCorrelation(
        assay(pe, "ptmMB"),
        model_designs[[i]]$designmatrix,
        block = pe$Extraction_batch
    )$consensus.correlation %>%
        {
            print(paste0("Model '", names(formulas)[[i]], "', extraction batch intra-block correlation = ", .[[1]]))
        }
}
```

```
[1] "Model 'factor', extraction batch intra-block correlation = 0.394110283189133"
[1] "Model 'timeseries_linear', extraction batch intra-block correlation = 0.382465046206923"
[1] "Model 'timeseries_quadratic', extraction batch intra-block correlation = 0.389511145371915"
```

Code

```
# add extraction batch as a random effect to the model formula
for (i in seq_along(formulas)) {
    formulas[[i]] <- paste0(formulas[[i]], " + (1|Extraction_batch)") %>%
        as.formula()
}
```

Now specify the relevant contrasts.

Code

```
# define the contrasts
contrasts <- list()
contrasts[["factor"]] <- c(
    "AFB1+ vs. DMSO+, 0h" = "Treat_EBVAFB1_Pos - Treat_EBVDMSO_Pos", # should be all 0
    "AFB1 vs. DMSO, 0h" = "Treat_EBVAFB1_Neg - Treat_EBVDMSO_Neg", # should be all 0
    "DMSO+ vs. DMSO, 0h" = "Treat_EBVDMSO_Pos - Treat_EBVDMSO_Neg",
    "AFB1+ vs DMSO+, 24h" = "(Treat_EBVAFB1_Pos + `Treat_EBVAFB1_Pos:as.factor(Time)24`) - (Treat_EBVDMSO_Pos + `Treat_EBVDMSO_Pos:as.factor(Time)24`)",
    "AFB1 vs DMSO, 24h" = "(Treat_EBVAFB1_Neg + `Treat_EBVAFB1_Neg:as.factor(Time)24`) - Treat_EBVDMSO_Neg",
    "AFB1+ vs DMSO+, 48h" = "(Treat_EBVAFB1_Pos + `Treat_EBVAFB1_Pos:as.factor(Time)48`) - (Treat_EBVDMSO_Pos + `Treat_EBVDMSO_Pos:as.factor(Time)48`)",
    "AFB1 vs DMSO, 48h" = "(Treat_EBVAFB1_Neg + `Treat_EBVAFB1_Neg:as.factor(Time)48`) - Treat_EBVDMSO_Neg",
    "AFB1+ vs DMSO+, 72h" = "(Treat_EBVAFB1_Pos + `Treat_EBVAFB1_Pos:as.factor(Time)72`) - (Treat_EBVDMSO_Pos + `Treat_EBVDMSO_Pos:as.factor(Time)72`)",
    "AFB1 vs DMSO, 72h" = "(Treat_EBVAFB1_Neg + `Treat_EBVAFB1_Neg:as.factor(Time)72`) - Treat_EBVDMSO_Neg",
    "AFB1+ (DMSO corrected) vs AFB1 (DMSO corrected) at 0h" = "(Treat_EBVAFB1_Pos - Treat_EBVDMSO_Pos) - (Treat_EBVAFB1_Neg - Treat_EBVDMSO_Neg)",
    "AFB1+ (DMSO corrected) vs AFB1 (DMSO corrected) at 24h" = "((Treat_EBVAFB1_Pos + `Treat_EBVAFB1_Pos:as.factor(Time)24`) - (Treat_EBVDMSO_Pos + `Treat_EBVDMSO_Pos:as.factor(Time)24`)) - ((Treat_EBVAFB1_Neg + `Treat_EBVAFB1_Neg:as.factor(Time)24`) - Treat_EBVDMSO_Neg)",
    "AFB1+ (DMSO corrected) vs AFB1 (DMSO corrected) at 48h" = "((Treat_EBVAFB1_Pos + `Treat_EBVAFB1_Pos:as.factor(Time)48`) - (Treat_EBVDMSO_Pos + `Treat_EBVDMSO_Pos:as.factor(Time)48`)) - ((Treat_EBVAFB1_Neg + `Treat_EBVAFB1_Neg:as.factor(Time)48`) - Treat_EBVDMSO_Neg)",
    "AFB1+ (DMSO corrected) vs AFB1 (DMSO corrected) at 72h" = "((Treat_EBVAFB1_Pos + `Treat_EBVAFB1_Pos:as.factor(Time)72`) - (Treat_EBVDMSO_Pos + `Treat_EBVDMSO_Pos:as.factor(Time)72`)) - ((Treat_EBVAFB1_Neg + `Treat_EBVAFB1_Neg:as.factor(Time)72`) - Treat_EBVDMSO_Neg)"
)
contrasts[["timeseries_linear"]] <- c(
    "DMSO+ vs. DMSO linear time effect"                                  = "Treat_EBVDMSO_Pos:Time - Treat_EBVDMSO_Neg:Time",
    "AFB1+ vs. DMSO+ linear time effect"                                 = "Treat_EBVAFB1_Pos:Time - Treat_EBVDMSO_Pos:Time",
    "AFB1 vs. DMSO linear time effect"                                   = "Treat_EBVAFB1_Neg:Time - Treat_EBVDMSO_Neg:Time",
    "AFB1+ (DMSO corrected) vs AFB1 (DMSO corrected) linear time effect" = "(Treat_EBVAFB1_Pos:Time - Treat_EBVDMSO_Pos:Time) - (Treat_EBVAFB1_Neg:Time - Treat_EBVDMSO_Neg:Time)"
)
contrasts[["timeseries_quadratic"]] <- c(
    "DMSO+ vs. DMSO linear time effect" = "`Treat_EBVDMSO_Pos:poly(Time, degree = 2)1` - `Treat_EBVDMSO_Neg:poly(Time, degree = 2)1`",
    "AFB1+ vs. DMSO+ linear time effect" = "`Treat_EBVAFB1_Pos:poly(Time, degree = 2)1` - `Treat_EBVDMSO_Pos:poly(Time, degree = 2)1`",
    "AFB1 vs. DMSO linear time effect" = "`Treat_EBVAFB1_Neg:poly(Time, degree = 2)1` - `Treat_EBVDMSO_Neg:poly(Time, degree = 2)1`",
    "AFB1+ (DMSO corrected) vs AFB1 (DMSO corrected) linear time effect" = "(`Treat_EBVAFB1_Pos:poly(Time, degree = 2)1` - `Treat_EBVDMSO_Pos:poly(Time, degree = 2)1`) - (`Treat_EBVAFB1_Neg:poly(Time, degree = 2)1` - `Treat_EBVDMSO_Neg:poly(Time, degree = 2)1`)",
    "DMSO+ vs. DMSO quadratic time effect" = "`Treat_EBVDMSO_Pos:poly(Time, degree = 2)2` - `Treat_EBVDMSO_Neg:poly(Time, degree = 2)2`",
    "AFB1+ vs. DMSO+ quadratic time effect" = "`Treat_EBVAFB1_Pos:poly(Time, degree = 2)2` - `Treat_EBVDMSO_Pos:poly(Time, degree = 2)2`",
    "AFB1 vs. DMSO quadratic time effect" = "`Treat_EBVAFB1_Neg:poly(Time, degree = 2)2` - `Treat_EBVDMSO_Neg:poly(Time, degree = 2)2`",
    "AFB1+ (DMSO corrected) vs AFB1 (DMSO corrected) quadratic time effect" = "(`Treat_EBVAFB1_Pos:poly(Time, degree = 2)2` - `Treat_EBVDMSO_Pos:poly(Time, degree = 2)2`) - (`Treat_EBVAFB1_Neg:poly(Time, degree = 2)2` - `Treat_EBVDMSO_Neg:poly(Time, degree = 2)2`)"
)
```

### 3.2 Statistical testing

Use MSqRob25 to fit robust linear mixed models for every protein, precursor, or PTM and perform testing.

Code

```
ridge_regression <- TRUE # flag for ease-of-use
# construct the contrast matrix
contrasts_full <- list()
L <- list()
for (i in seq_along(formulas)) {
    if (ridge_regression) {
        # prepend "ridge" to model parameters names to match output from msqrob function
        parameterNames <- paste0("ridge", colnames(model_designs[[i]]$designmatrix))
        # also do the above for the contrasts using regex matches to model parameter names
        # first escape all special characters from parameters to prevent the later regex from not matching
        regex_safe_parnames <- colnames(model_designs[[i]]$designmatrix) %>%
            # longest param first to prevent matching substring e.g. interaction terms
            {
                .[order(nchar(.), ., decreasing = TRUE)]
            } %>%
            str_replace_all(r"---{([\[\]\\*?+(){}^$|.])}---", r"---{\\\1}---") %>%
            {
                paste0("(", paste0(., collapse = "|"), ")")
            }
        contrasts_full[[i]] <- str_replace_all(
            contrasts[[i]],
            regex_safe_parnames,
            "ridge\\1"
        )
    } else {
        parameterNames <- colnames(model_designs[[i]]$designmatrix)
        contrasts_full[[i]] <- contrasts[[i]]
    }
    L[[i]] <- makeContrast(sapply(contrasts_full[[i]], paste, "= 0"), parameterNames = parameterNames)
}
# lists to store dataframes
hypothesis_tests <- list()
signif_pval_thresh <- list()
for (viz_assay in c("co-extracts", "variantMB", "precursorMB", "ptmMB")) {
    for (i in seq_along(formulas)) {
        # model fitting and hypothesis tests using MSqRob2
        pe <- msqrob2::msqrob(
            object = pe,
            i = viz_assay,
            formula = formulas[[i]],
            modelColumnName = names(formulas)[[i]],
            robust = TRUE,
            maxitRob = 20,
            ridge = ridge_regression,
            overwrite = TRUE
        ) %>%
            hypothesisTest(
                i = viz_assay,
                contrast = L[[i]],
                modelColumn = names(formulas)[[i]],
                overwrite = TRUE
            )
        # extract hypothesis test results
    }
    hypothesis_tests[[viz_assay]] <- do.call(rbind, rowData(pe[[viz_assay]])[, unlist(contrasts_full)]) %>%
        as_tibble() %>%
        mutate(
            contrast = {
                sapply(contrasts, names) %>%
                    unlist(use.names = FALSE) %>%
                    rep(each = nrow(pe[[viz_assay]])) %>%
                    factor(levels = unlist(sapply(contrasts, names))[!duplicated(unlist(sapply(contrasts, names)))])
            },
            name = if (viz_assay == "precursorMB") {
                rep.int(rowData(pe[[viz_assay]])$precursor, length(unlist(contrasts)))
            } else {
                rep.int(rownames(pe[[viz_assay]]), length(unlist(contrasts)))
            },
            family = rep.int(rowData(pe[[viz_assay]])$family, length(unlist(contrasts))),
            formula = rep(names(formulas), times = sapply(contrasts, length) * nrow(pe[[viz_assay]]))
        )
    # calculate BH threshold for each contrast using (rank of first non-significant test
    # / total number of tests * FDR)
    signif_pval_thresh[[viz_assay]] <- hypothesis_tests[[viz_assay]] %>%
        group_by(contrast, formula) %>%
        summarise(BH_thresh = (sum(adjPval < 0.05, na.rm = TRUE) + 1) / n() * 0.05, .groups = "drop")
}
```

And present the significant differences for every contrast:

Code

```
assay_names <- list(
    "co-extracts" = "All proteins",
    "variantMB" = "Histone variants",
    "precursorMB" = "Histone precursors",
    "ptmMB" = "Histone PTMs"
)

volcanos <- list()
volcano_layout <- list()
for (viz_assay in c("co-extracts", "variantMB", "precursorMB", "ptmMB")) {
    for (formula_name in names(formulas)) {
        # static plot
        volcanos[[viz_assay]][[formula_name]] <- hypothesis_tests[[viz_assay]] %>%
            filter(formula == formula_name) %>%
            ggplot(
                aes(
                    x = logFC,
                    y = -log10(pval),
                    color = adjPval < 0.05,
                    shape = if (viz_assay == "co-extracts") {
                        NULL
                    } else {
                        family
                    },
                    text = if (viz_assay == "co-extracts") {
                        name
                    } else {
                        paste(family, name, sep = "#")
                    }
                )
            ) +
            geom_point() +
            geom_hline(
                data = filter(signif_pval_thresh[[viz_assay]], formula == formula_name),
                aes(yintercept = -log10(BH_thresh)),
                linetype = "dashed",
                color = "#c44133ff"
            ) +
            geom_vline(xintercept = 0, linetype = "dashed", color = "#253544ff") +
            facet_wrap(vars(contrast)) +
            theme_bw() +
            theme(plot.title = element_text(face = "bold")) +
            scale_color_manual(values = alpha(c("#253544ff", "#c44133ff"))) +
            coord_cartesian(xlim = c(-max(abs(filter(hypothesis_tests[[viz_assay]], formula == formula_name)$logFC)), max(abs(filter(hypothesis_tests[[viz_assay]], formula == formula_name)$logFC))))
        # print(volcanos[[viz_assay]][[formula_name]] +
        #     labs(
        #         x = bquote(log[2](FC)),
        #         y = bquote(-log[10](Pval)),
        #         color = bquote(Pval[BH] < 0.05),
        #         shape = "Histone family",
        #         title = paste0("Model ", formula_name, ", ", assay_names[[viz_assay]])
        #     ))
        volcano_layout[[formula_name]] <- ggplot_build(volcanos[[viz_assay]][[formula_name]])$layout$layout # for output width/height
        ggsave(
            plot = volcanos[[viz_assay]][[formula_name]] +
                labs(
                    x = bquote(log[2](FC)),
                    y = bquote(-log[10](Pval)),
                    color = bquote(Pval[BH] < 0.05),
                    shape = "Histone family",
                    title = paste0("Model ", formula_name, ", ", assay_names[[viz_assay]])
                ),
            filename = paste0(
                "./out/floats/",
                str_replace_all(assay_names[[viz_assay]], fixed(" "), "_"),
                "_",
                formula_name,
                "_volcano.pdf"
            ), width = 40 + 50 * max(volcano_layout[[formula_name]]$COL),
            height = 60 * max(volcano_layout[[formula_name]]$ROW),
            units = "mm", dpi = 300
        )
    }
}
```

- Model factor, All proteins
- Model timeseries\_linear, All proteins
- Model timeseries\_quadratic, All proteins
- Model factor, Histone variants
- Model timeseries\_linear, Histone variants
- Model timeseries\_quadratic, Histone variants
- Model factor, Histone precursors
- Model timeseries\_linear, Histone precursors
- Model timeseries\_quadratic, Histone precursors
- Model factor, Histone PTMs
- Model timeseries\_linear, Histone PTMs
- Model timeseries\_quadratic, Histone PTMs

Finally, we can write out the volcano plots and results

Code

```
# write the results from differential abundance/usage analysis
for (viz_assay in c("co-extracts", "variantMB", "precursorMB", "ptmMB")) {
    # export results tables
    hypothesis_tests[[viz_assay]] %>%
        arrange(pval) %>%
        group_by(formula, contrast) %>%
        mutate(within_formula_contrast_rank = order(order(pval))) %>%
        ungroup() %>%
        mutate(
            signif = adjPval < 0.05,
            across(where(is.numeric), ~ signif(., 3))
        ) %>%
        write_csv(file = paste0("./out/floats/contrast_", viz_assay, ".csv"))
}
archive::archive_write_dir("./out/other/contrasts.zip", "./out/floats/")
```

The results of the differential abundance analysis can be downloaded here:

Download results

## Footnotes

1. Demeulemeester, N., Gébelin, M., Caldi Gomes, L., Lingor, P., Carapito, C., Martens, L., & Clement, L. (2024). msqrob2PTM: Differential Abundance and Differential Usage Analysis of MS-Based Proteomics Data at the Posttranslational Modification and Peptidoform Level. Molecular & Cellular Proteomics, 23(2), 100708. https://doi.org/10.1016/j.mcpro.2023.100708↩︎
2. Nonlinear Dynamics, Waters. Progenesis QI for proteomics (Version 4.2). https://www.nonlinear.com/progenesis/qi-for-proteomics/↩︎
3. Gatto, L., & Vanderaa, C. (2024). QFeatures: Quantitative features for mass spectrometry data (Version 1.16.0) [R]. Release (3.20). https://doi.org/10.18129/B9.bioc.QFeatures↩︎
4. Huber, W., von Heydebreck, A., Sültmann, H., Poustka, A., & Vingron, M. (2002). Variance stabilization applied to microarray data calibration and to the quantification of differential expression. Bioinformatics, 18(suppl\_1), S96–S104. https://doi.org/10.1093/bioinformatics/18.suppl\_1.S96↩︎
5. Goeminne, LudgerJ. E., Gevaert, K., & Clement, L. (2016). Peptide-level Robust Ridge Regression Improves Estimation, Sensitivity, and Specificity in Data-dependent Quantitative Label-free Shotgun Proteomics \*. Molecular & Cellular Proteomics, 15(2), 657–668. https://doi.org/10/f78fjf↩︎


##### Source Code

```
---
title: "Differential hPTM usage"
subtitle: EpiMycoTox — Thanos Mouchtaris-Michailidis
---

## Overview

This workflow is an adaptation of msqrob2PTM^[Demeulemeester, N., Gébelin, M., Caldi Gomes, L., Lingor, P., Carapito, C., Martens, L., & Clement, L. (2024). msqrob2PTM: Differential Abundance and Differential Usage Analysis of MS-Based Proteomics Data at the Posttranslational Modification and Peptidoform Level. Molecular & Cellular Proteomics, 23(2), 100708. https://doi.org/10.1016/j.mcpro.2023.100708].
In brief, data is imported, filtered, and normalized in two steps (general and usage
normalization). A peptide ion data export from Progenesis QIP^[Nonlinear Dynamics, Waters. Progenesis QI for proteomics (Version 4.2). https://www.nonlinear.com/progenesis/qi-for-proteomics/]
is preprocessed in @sec-preprocessing and differential abundance analysis is then
performed in @sec-da.

We start by importing the relevant packages.

```{r}
#| label: chunk-libraries
#| output: false

# tidyverse
library(tidyverse)
# proteomics
library(QFeatures)
library(msqrob2)
# custom functions
source("./src/consensus_hPTM_utils.R")
```

## Data preprocessing {#sec-preprocessing}

### Data import

First, read in the hPTM dataset (all peptide ion export from Progenesis QIP) and
transform into a QFeatures^[Gatto, L., & Vanderaa, C. (2024). QFeatures: Quantitative features for mass spectrometry data (Version 1.16.0) [R]. Release (3.20). https://doi.org/10.18129/B9.bioc.QFeatures]
object. This data contains all identified peptide ions with their lfq abundances per
run, histone features being feature edited and tagged in Progenesis QIP.

```{r}
#| label: chunk-import
#| column: page-right

# read the histone dataset
quantcol_pattern <- "231030_"
df_histone <- read_csv("./data/processed/241202_peptide_export_all_proteins_parsed.csv", col_types = cols(
    feat = col_integer(),
    protein = col_character(),
    histone = col_logical(),
    contaminant = col_logical(),
    sequence = col_character(),
    charge = col_integer(),
    mods = col_character(),
    .default = col_double()
)) %>%
    mutate(
        precursor = paste(sequence, charge, mods, sep = "_"),
        protein = str_replace_all(protein, ";", "/"),
        family = str_extract(protein, "^H(1|2A|2B|3|4)"),
        core = grepl("^H(2A|2B|3|4)", family)
    )
# read metadata
colData <- read_csv("./data/raw/epimycotox_thanos_metadata.csv", col_types = cols(
    Sample_name = col_character(),
    Include = col_logical(),
    Outlier = col_logical(),
    Treatment = col_factor(),
    EBV = col_factor(),
    Time = col_integer(),
    Replicate = col_factor(),
    Extraction_batch = col_factor()
))
# check for mismatch between samples in quantitative dataset and metadata
common_samples <- intersect(colnames(df_histone), colData$Sample_name)
quant_not_meta <- setdiff(grep(quantcol_pattern, colnames(df_histone), value = TRUE), colData$Sample_name)
if (length(quant_not_meta) > 0) {
    stop(sprintf(
        "Samples in quantitative data but not in metadata, add to metadata .csv: %s",
        paste(quant_not_meta, collapse = ", ")
    ))
}
meta_not_quant <- setdiff(colData$Sample_name, colnames(df_histone))
if (length(meta_not_quant) > 0) {
    dropped <- paste(meta_not_quant, collapse = ", ")
    colData <- colData[colData$Sample_name %in% common_samples, ]
}
# simplify sample names and specify groups
df_histone <- rename_with(
    df_histone,
    str_replace_all,
    pattern = paste0(quantcol_pattern, "|_02"),
    replacement = ""
)
colData <- arrange(colData, match(common_samples, Sample_name)) %>%
    mutate(quantCols = str_replace_all(
        Sample_name,
        pattern = paste0(quantcol_pattern, "|_02"),
        replacement = ""
    ), .keep = "unused") %>%
    mutate(
        Group = paste(Treatment, EBV, Time, sep = "_"),
        Treat_EBV = paste(Treatment, EBV, sep = "_"),
        across(c(Group, Treat_EBV), as.factor)
    )
# to QFeatures object
pe <- readQFeatures(
    df_histone,
    colData = colData,
    name = "precursorRaw",
    verbose = FALSE
)
if (length(meta_not_quant) > 0) {
    print(sprintf(
        "Samples in metadata but not in quantitative data, dropping: %s",
        str_replace_all(dropped, pattern = quantcol_pattern, replacement = "")
    ))
}
```

Inspect if the following looks correct:

```{r}
#| label: tbl-import
#| panel: tabset
#| results: asis
#| echo: false
#| column: page-right

cat("\n#### Precursor metadata\n\n")
as_tibble(rowData(pe[["precursorRaw"]]))
cat("\n#### Sample metadata\n\n")
as_tibble(colData(pe))
```

### Sample QC

Next, we can inspect the peptide loading consistency and histone extraction efficiency.

```{r}
#| label: fig-extraction
#| fig-cap: "Evaluation of histone loading per extraction batch"
#| message: false
#| warning: false

# sample loading
nonhistone_assay <- assay(pe[!rowData(pe[["precursorRaw"]])$histone, ], "precursorRaw")
median_raw_nonhistone_abundance <- colMedians(log2(nonhistone_assay), na.rm = TRUE)
colData(pe)[names(median_raw_nonhistone_abundance), "median_raw_nonhistone_abundance"] <- median_raw_nonhistone_abundance
# histone loading
histone_assay <- assay(pe[rowData(pe[["precursorRaw"]])$histone, ], "precursorRaw")
median_raw_histone_abundance <- colMedians(log2(histone_assay), na.rm = TRUE)
colData(pe)[names(median_raw_histone_abundance), "median_raw_histone_abundance"] <- median_raw_histone_abundance
# visualize loading consistency / histone extraction efficiency
p <- ggplot(
    colData(pe[, !grepl("QC_", pe$quantCols)]),
    aes(x = median_raw_histone_abundance, y = median_raw_nonhistone_abundance, color = Extraction_batch, shape = Outlier)
) +
    geom_point() +
    geom_smooth(aes(color = NULL, shape = NULL), se = FALSE) +
    geom_abline(intercept = 0, slope = 1)
p <- ggExtra::ggMarginal(p, groupFill = TRUE, position = "stack")
grid::grid.newpage()
grid::grid.draw(p)
```

Clear differences in extraction efficiency can be seen between extraction batches. This
will need to be corrected for during modelling. Some outlier samples will be removed
although nothing extreme.

### Filtering

Remove precursors with less than 75 % presence.

```{r}
#| label: chunk-filtering
#| message: false

# drop outliers
pe <- pe[, !pe$Outlier]
rows_before_filter <- nrow(pe[["precursorRaw"]])
# drop contaminant features
pe <- filterFeatures(pe, ~ !contaminant)
# remove high missing features
pe <- zeroIsNA(pe, i = "precursorRaw")
pe_nNA <- nNA(pe, i = "precursorRaw")
# at least 3 samples should have the precursor quantified + 75 % across
pe <- pe[!(ncols(pe)[[1]] - pe_nNA$nNArows$nNA < 3 | pe_nNA$nNArows$pNA > 0.25), ] %>%
    # simplify protein grouping, for co-extracts?
    filterFeatures(~ protein %in% smallestUniqueGroups(protein, split = "/"))
```

This leaves us with `r nrow(pe[["precursorRaw"]])` observations (`r rows_before_filter - nrow(pe[["precursorRaw"]])`
rows dropped). We can now look at the histone family coverage.

```{r}
#| label: fig-families
#| fig-cap: "Number of unique precursors (peptide + charge + modifications if any) quantified per histone family"

# plot remaining
rowData(pe[rowData(pe[["precursorRaw"]])$histone, ][["precursorRaw"]]) %>%
    ggplot(aes(
        x = family,
        fill = !is.na(mods) & !sapply(str_split(mods, ";"), function(x) all(grepl("Unmod", x)))
    )) +
    geom_bar() +
    labs(fill = "Modified")
```

### Normalization

Normalization of the samples is performed in two steps:

1. Removal of technical variation, such as from sample loading differences, using
variance stabilization (VSN)^[Huber, W., von Heydebreck, A., Sültmann, H., Poustka, A., & Vingron, M. (2002). Variance stabilization applied to microarray data calibration and to the quantification of differential expression. Bioinformatics, 18(suppl_1), S96–S104. https://doi.org/10.1093/bioinformatics/18.suppl_1.S96].
This normalizes all samples based on the abundance distribution of all precursors
2. Correction of histone variant/nucleosome abundance differences to allow differential
**usage** analysis of histone variants and PTMs.

#### Global normalization

Start with VSN normalization and evaluate the results.

::: {.column-page}

```{r}
#| label: fig-vsn
#| fig-cap: "Evaluation of global normalization"
#| fig-subcap:
#|   - "MeanSdPlot for quality check of VSN normalization: the red line should be approximately horizontal"
#|   - "Density plot of normalized precursor abundances per sample, coloured by extraction batch"
#|   - "Boxplot of normalized precursor abundances per sample, coloured by extraction batch"
#| layout-ncol: 3
#| column: page
#| message: false

# VSN requires the non-log transformed values as it glog transforms the data
pe <- normalize(pe, i = "precursorRaw", method = "vsn", name = "precursor", verbose = FALSE)
# normalized histone abundances
histone_assay <- assay(pe, "precursor")
median_histone_abundance <- colMedians(histone_assay, na.rm = TRUE)
colData(pe)[names(median_histone_abundance), "median_histone_abundance"] <- median_histone_abundance

# visualize
vsn::meanSdPlot(assay(pe[["precursor"]]))
d <- limma::plotDensities(assay(pe[["precursor"]]), col = as.numeric(pe$Group), legend = FALSE)
boxplot(assay(pe[["precursor"]]), col = pe$Group, ylab = "Intensity")
```

One sample (`r pe$quantCols[which.max(apply(d$Y, 2, max))]`) looks different from the
rest. This sample will also be discarded.

```{r}
#| label: fig-vsn2
#| fig-cap: "Evaluation of global normalization after outlier removal"

pe <- pe[, -which.max(apply(d$Y, 2, max))]
limma::plotDensities(assay(pe[["precursor"]]), col = as.numeric(pe$Group), legend = FALSE)
```

:::

Peptide-level MDS plots, color-coded according to the metadata, will show the
level of similarity between samples. Look for any obvious clustering, if present. Do
note that this is based on all peptide data, including from co-extracts.

```{r}
#| label: chunk-plotlyinit
#| include: false
#| echo: false
#|
htmltools::tagList(plotly::plot_ly()) # initialize plot_ly for tabsets
```

```{r}
#| panel: tabset
#| results: asis

# MDS calculations
MDS_assay <- limma::plotMDS(assay(pe[["precursor"]]), plot = FALSE) %$%
    data.frame("Dim1" = x, "Dim2" = y, "var.explained" = var.explained) %>%
    cbind(colData(pe))
# plot
tabs <- colnames(colData(pe))[!colnames(colData(pe)) %in% c("Outlier", "Treat_EBV", "Group", "quantCols")]
for (fill_col in tabs) {
    cat("#### ", fill_col, "\n\n")
    p <- ggplot(MDS_assay, mapping = aes(
        x = Dim1, y = Dim2, fill = .data[[fill_col]],
        size = pe$median_histone_abundance, annotation = rownames(MDS_assay)
    )) +
        geom_point(colour = "black", shape = 21) +
        theme_bw() +
        xlab(paste("Leading logFC dim 1 (", round(100 * MDS_assay$var.explained[[1]], 2), "%)", sep = "")) +
        ylab(paste("Leading logFC dim 2 (", round(100 * MDS_assay$var.explained[[2]], 2), "%)", sep = ""))
    p %>%
        plotly::ggplotly() %>%
        htmltools::tagList() %>%
        print()
    cat("\n\n")
}
```

From the MDS plots, we can see that the extraction batch (clear clusters) and loading
differences (median histone abundance ~ PC2) are the main drivers of dissimilarity
between samples, even after normalization. We will need to take this into account during
the modelling step.

We can already aggregate the co-extracts to the protein level for later testing.

```{r}
#| label: chunk-protein-agg
#| message: false
#| warning: false

# select co-extracts and do a second mode between protein fold change normalization, as per MS-DAP
pe <- addAssay(pe, subset(pe[["precursor"]], subset = !histone), "precursorCoextr") %>%
    addAssayLink("precursor", "precursorCoextr", "feat", "feat") %>%
    normalize_usage(
        i = "precursorCoextr",
        fcol = "protein",
        name = "precursorCoextrMB",
        groups = "Group",
        scaling_fun = "FCBM",
        aggregation_fun = MsCoreUtils::robustSummary
    )
# aggregate co-extracts
pe <- aggregateFeatures(
    pe,
    i = "precursorCoextrMB",
    fcol = "protein",
    name = "co-extracts",
    fun = MsCoreUtils::robustSummary
)
```

#### Usage normalization

To perform the second normalization step, we first create a histone assay in which we
deconvolute the data to the single PTM level.

```{r}
#| label: chunk-deconvolution
#| message: false

# deconvolute histone peptidoforms based on sPTM definition
pe <- deconvolute(
    pe,
    i = "precursor",
    fcol = "precursor",
    deconv = "mods",
    top_level = "protein",
    name = "precursorDeconv",
    filter = ~histone
)
```

A total of `r length(unique(rowData(pe[["precursorDeconv"]])$deconvoluted))` single
PTMs were defined. Now we perform the second normalization step. We do this by centering
the fold change differences between sample groups at both the histone variant and PTM
level, i.e., fold change distributions for histone variant and PTM abundance between two
sample groups should center around zero.

```{r}
#| label: chunk-fcmb
#| message: false
#| warning: false

# normalize histone peptidoforms
pe <- normalize_usage(
    pe,
    i = c("precursorMods", "precursorDeconv"),
    fcol = list("precursorMods" = "family", "precursorDeconv" = "deconvoluted"),
    name = c("precursorMB", "precursorDeconvMB"),
    combine_fcol = TRUE,
    groups = NULL,
    sep_level = "core",
    scaling_fun = "FCBM",
    aggregation_fun = MsCoreUtils::robustSummary
)
```

Now aggregate to the single PTM level, starting from the relevant assays, and perform
some QC checks.

```{r}
#| label: fig-fcmb
#| fig-cap: "Distribution of pairwise logFC modes between sample groups (gray) and their
#|           overall mean distribution (purple)."
#| fig-subcap:
#|      - "Protein-level, before usage normalization"
#|      - "Protein-level, before usage normalization"
#|      - "PTM-level, before usage normalization"
#|      - "PTM-level, after usage normalization"
#| layout-ncol: 2
#| message: false
#| warning: false

# helper function
as_matrix <- function(x) {
    y <- as.matrix.data.frame(x[, -1])
    rownames(y) <- x[[1]]
    y
}

logfc_means <- list()
shapiro_test <- list()
# every entry of the list will be one assay to aggregate and check between-group logFC modes
for (viz_assay in list(
    c("precursorMods", "protein", "variant_noMB"),
    c("precursorMB", "protein", "variantMB"),
    c("precursorDeconv", "deconvoluted", "ptm_noMB"),
    c("precursorDeconvMB", "deconvoluted", "ptmMB")
)) {
    # calculate pairwise logFC from assay
    pe <- aggregateFeatures(
        pe,
        i = viz_assay[[1]],
        fcol = viz_assay[[2]],
        name = viz_assay[[3]],
        fun = MsCoreUtils::robustSummary, # consider medianPolish if this is too slow, scales better
        na.rm = TRUE
    )
    # calculate between-group logfc modes
    logfc_modes <- assay(pe, viz_assay[[3]]) %>%
        t() %>%
        as_tibble() %>%
        group_by(pe$Group) %>%
        summarise(across(everything(), ~ mean(.x, na.rm = TRUE))) %>%
        as_matrix() %>%
        t() %>%
        pairwise_modes()
    # rowmedians and normality test for every pairwise logfc comparison
    logfc_means[[viz_assay[[3]]]] <- rowMeans(logfc_modes)
    shapiro_test[[viz_assay[[3]]]] <- apply(logfc_modes, 1, shapiro.test)
    # density plot
    limma::plotDensities(logfc_modes,
        legend = FALSE, col = "darkgray",
        main = paste("Pairwise between-group LogFC, assay", viz_assay[[3]])
    )
    # add overall mean density to plot
    d <- apply(logfc_modes, 1, function(x) {
        density(x,
            bw = "SJ", from = range(logfc_modes)[[1]],
            to = range(logfc_modes)[[2]], na.rm = TRUE
        )
    })
    matlines(d[[1]]$x, rowMeans(sapply(d, function(x) x$y)), lwd = 6, col = "darkviolet")
    abline(v = 0, col = "gray20", lty = "dashed", lwd = 2)
}
```

LogFC differences between sample groups center well around 0, more so at the PTM level
than at the histone variant level (more ambiguity, so not unexpected). Usage
normalization decreased the spread of these distributions, correcting for "nucleosome
abundance" differences between samples.

```{r}
#| label: tbl-shapiro
#| tbl-cap: "Summary statistics of pairwise logFC mode between sample calculations at
#|           both the histone variant and PTM level."
#| tbl-subcap:
#|      - "Absolute mean logFC modes between sample groups before usage normalization,
#|         sorted in decreasing order. Higher values signify large differences compared
#|         to other sample groups, technical (e.g. low histone extraction yield) or
#|         biological (e.g. high cell death, ...)."
#|      - "Shapiro-Wilk p values of pairwise logFC mode distributions between sample
#|         groups after usage normalization, sorted by increasing mean. Low p values
#|         mean more evidence against normality; sample groups with low p values may be
#|         highly differential/outliers."
#| layout-ncol: 1

as_tibble(logfc_means[c("variant_noMB", "ptm_noMB")]) %>%
    mutate(row_mean = abs(rowMeans(across(everything())))) %>%
    mutate(across(everything(), ~ signif(., 4))) %>%
    mutate(group = names(logfc_means[[1]]), .before = 1) %>%
    arrange(desc(row_mean))

lapply(shapiro_test[c("variantMB", "ptmMB")], function(x) {
    sapply(x, function(y) y$p)
}) %>%
    as_tibble() %>%
    mutate(row_mean = rowMeans(across(everything()))) %>%
    mutate(across(everything(), ~ signif(., 4))) %>%
    mutate(group = names(shapiro_test[[1]]), .before = 1) %>%
    arrange(row_mean)
```

Now we can repeat the MDS analysis, but instead of the globally normalized peptide-level
data we now only use histone data at different assay levels:

* Left, usage normalized **precursor** level
* Middle, usage normalized **single PTM** level through robust summarization from deconvoluted precursor level
* Right, usage normalized **histone variant** level through robust summarization from precursor level

::: {.column-page}

MDS plots of all samples after general and usage normalization:

::: {.panel-tabset}

```{r}
#| results: asis

MDS_assays <- list()
for (viz_assay in c("precursorMB", "variantMB", "ptmMB")) {
    MDS_assay <- limma::plotMDS(assay(pe, viz_assay), plot = FALSE) %$%
        data.frame("Dim1" = x, "Dim2" = y, "var.explained" = var.explained) %>%
        cbind(colData(pe)) %>%
        mutate(assay = viz_assay)
    MDS_assays[[viz_assay]] <- MDS_assay
}
MDS_assays <- do.call(rbind, MDS_assays)
# plot
tabs <- colnames(colData(pe))[!colnames(colData(pe)) %in% c("Outlier", "Group", "quantCols")]
for (fill_col in tabs) {
    cat("#### ", fill_col, "\n\n")
    p <- ggplot(MDS_assays, mapping = aes(
        x = Dim1, y = Dim2, fill = .data[[fill_col]],
        size = median_histone_abundance, annotation = rownames(MDS_assays)
    )) +
        geom_point(colour = "black", shape = 21) +
        facet_wrap(~assay, nrow = 1, scales = "free") +
        theme_bw() +
        xlab(paste("Leading logFC dim 1 (", round(100 * MDS_assay$var.explained[[1]], 2), "%)", sep = "")) +
        ylab(paste("Leading logFC dim 2 (", round(100 * MDS_assay$var.explained[[2]], 2), "%)", sep = ""))
    p %>%
        plotly::ggplotly() %>%
        htmltools::tagList() %>%
        print()
    cat("\n\n")
}
```

:::

:::

The extraction batch remains the main driver of dissimilarity between samples. No other
clusters are apparent. Finally, the preprocessed dataset and all its assays can be
represented as such:

```{r}
#| label: fig-qfeatures
#| fig-cap: "Visual representation of the QFeatures object holding all data and its relations"
#| echo: False

plot(pe)
```

## Differential usage analysis {#sec-da}

### Model building

We will start by creating the model formula and relevant contrasts, then we can proceed
with differential usage testing. First, how should we model the time effect?

::: {.panel-tabset group="assay"}

```{r}
#| results: asis
#| message: False
#| warning: False

# create long format dataframe for ggplot
peptidoform <- QFeatures::longFormat(
    pe[, , c("precursorMB")],
    colvars = c("Time", "Treat_EBV"),
    rowvars = c("precursor", "protein", "family"),
    index = 1L
) %>%
    as_tibble() %>%
    mutate(ID = precursor, .keep = "unused") %>%
    select(-rowname)
aggregated <- QFeatures::longFormat(
    pe[, , c("variantMB", "ptmMB")],
    colvars = c("Time", "Treat_EBV"),
    rowvars = c("protein", "family"),
    index = 1L
) %>%
    as_tibble() %>%
    mutate(ID = rowname, .keep = "unused")
pe_long <- rbind(peptidoform, aggregated) %>%
    mutate(
        grouper = paste(assay, Treat_EBV, protein, ID, sep = "_"),
        grouper_time = paste0(grouper, Time)
    )
# calculate means for every unique ID and time + drop IDs with only 1 timepoints
group_time_means <- pe_long %>%
    group_by(grouper, Time) %>%
    summarise(mean = mean(value, na.rm = TRUE)) %>%
    mutate(grouper_time = paste0(grouper, Time))
timeseries_groups <- group_time_means %>%
    summarise(count = n()) %>%
    filter(count > 1)
pe_long <- pe_long[pe_long$grouper %in% timeseries_groups$grouper, ]
pe_long["mean"] <- group_time_means[match(pe_long$grouper_time, group_time_means$grouper_time), ]$mean
# # start all lines at 0
t0 <- pe_long[pe_long$Time == 0, ]
for (level in unique(pe_long$Time)) {
    t_current <- pe_long[pe_long$Time == level, ]
    # make sure the order is preserved
    t0 <- t0[match(t_current$grouper, t0$grouper), ]
    pe_long[pe_long$Time == level, "mean"] <- t_current$mean - t0$mean
}
# plot
for (viz_assay in c("variantMB", "precursorMB", "ptmMB")) {
    cat("#### ", viz_assay, "\n\n")
    p <- pe_long %>%
        drop_na(mean) %>%
        filter(assay == viz_assay) %>%
        ggplot(aes(x = Time, y = mean, group = grouper, col = family)) +
        geom_line(alpha = .4) +
        facet_wrap(~Treat_EBV)
    p %>%
        plotly::ggplotly(tooltip = c("protein", "Time", "mean", "grouper")) %>%
        htmltools::tagList() %>%
        print()
    cat("\n\n")
}
```

:::

As there is only one timepoint of VPA, we cannot include these samples in the timeseries
design. We will make three models: one "means model" with time as a factor, one
"regression model" with linear time as a covariate, and one regression model with
quadratic time as a covariate.

```{r}
#| label: fig-designmat
#| fig-cap: "Visualization of the factor design matrix."
#| layout-ncol: 1

# drop runs not in the "Include" column of colData, e.g., QC runs or outliers
# also drop VPA samples, as we only have 1 timepoint for these
pe <- pe[, pe$Include & !pe$Treatment == "VPA"]
colData(pe) <- droplevels(colData(pe))
# relevel factors as needed
pe$Treatment <- relevel(pe$Treatment, "DMSO")
pe$Treat_EBV <- relevel(pe$Treat_EBV, "DMSO_Neg")
# establish the correct formula and explore the design, do not yet include random effects
# ExploreModelMatrix::ExploreModelMatrix(colData(pe)[, !apply(colData(pe), 2, function(x) any(is.na(x)))])
formulas <- list()
formulas[["factor"]] <- "~ 0 + Treat_EBV * as.factor(Time)"
formulas[["timeseries_linear"]] <- "~ 0 + EBV + Treat_EBV:Time"
formulas[["timeseries_quadratic"]] <- "~ 0 + EBV + Treat_EBV:poly(Time, degree=2)"
model_designs <- list()
for (designFormula in formulas) {
    model_designs[[designFormula]] <- ExploreModelMatrix::VisualizeDesign(
        colData(pe),
        designFormula = as.formula(designFormula),
        textSizeFitted = 2
    )
    if (designFormula == "~ 0 + Treat_EBV * as.factor(Time)") { # only really informative for factor model
        purrr::walk(model_designs[[designFormula]]$plotlist, print)
    }
}
```

We can include other model parameters to account for random variation: typical examples
are loading differences or sample preparation batches. For random effects, we can
estimate the intra-block correlation to determine whether or not to include these as
random effects.

```{r}
#| label: chunk-random-effects

# check if we should include random effects
for (i in seq_along(formulas)) {
    limma::duplicateCorrelation(
        assay(pe, "ptmMB"),
        model_designs[[i]]$designmatrix,
        block = pe$Extraction_batch
    )$consensus.correlation %>%
        {
            print(paste0("Model '", names(formulas)[[i]], "', extraction batch intra-block correlation = ", .[[1]]))
        }
}
# add extraction batch as a random effect to the model formula
for (i in seq_along(formulas)) {
    formulas[[i]] <- paste0(formulas[[i]], " + (1|Extraction_batch)") %>%
        as.formula()
}
```

Now specify the relevant contrasts.

```{r}
#| label: chunk-contrasts
#| code-fold: show

# define the contrasts
contrasts <- list()
contrasts[["factor"]] <- c(
    "AFB1+ vs. DMSO+, 0h" = "Treat_EBVAFB1_Pos - Treat_EBVDMSO_Pos", # should be all 0
    "AFB1 vs. DMSO, 0h" = "Treat_EBVAFB1_Neg - Treat_EBVDMSO_Neg", # should be all 0
    "DMSO+ vs. DMSO, 0h" = "Treat_EBVDMSO_Pos - Treat_EBVDMSO_Neg",
    "AFB1+ vs DMSO+, 24h" = "(Treat_EBVAFB1_Pos + `Treat_EBVAFB1_Pos:as.factor(Time)24`) - (Treat_EBVDMSO_Pos + `Treat_EBVDMSO_Pos:as.factor(Time)24`)",
    "AFB1 vs DMSO, 24h" = "(Treat_EBVAFB1_Neg + `Treat_EBVAFB1_Neg:as.factor(Time)24`) - Treat_EBVDMSO_Neg",
    "AFB1+ vs DMSO+, 48h" = "(Treat_EBVAFB1_Pos + `Treat_EBVAFB1_Pos:as.factor(Time)48`) - (Treat_EBVDMSO_Pos + `Treat_EBVDMSO_Pos:as.factor(Time)48`)",
    "AFB1 vs DMSO, 48h" = "(Treat_EBVAFB1_Neg + `Treat_EBVAFB1_Neg:as.factor(Time)48`) - Treat_EBVDMSO_Neg",
    "AFB1+ vs DMSO+, 72h" = "(Treat_EBVAFB1_Pos + `Treat_EBVAFB1_Pos:as.factor(Time)72`) - (Treat_EBVDMSO_Pos + `Treat_EBVDMSO_Pos:as.factor(Time)72`)",
    "AFB1 vs DMSO, 72h" = "(Treat_EBVAFB1_Neg + `Treat_EBVAFB1_Neg:as.factor(Time)72`) - Treat_EBVDMSO_Neg",
    "AFB1+ (DMSO corrected) vs AFB1 (DMSO corrected) at 0h" = "(Treat_EBVAFB1_Pos - Treat_EBVDMSO_Pos) - (Treat_EBVAFB1_Neg - Treat_EBVDMSO_Neg)",
    "AFB1+ (DMSO corrected) vs AFB1 (DMSO corrected) at 24h" = "((Treat_EBVAFB1_Pos + `Treat_EBVAFB1_Pos:as.factor(Time)24`) - (Treat_EBVDMSO_Pos + `Treat_EBVDMSO_Pos:as.factor(Time)24`)) - ((Treat_EBVAFB1_Neg + `Treat_EBVAFB1_Neg:as.factor(Time)24`) - Treat_EBVDMSO_Neg)",
    "AFB1+ (DMSO corrected) vs AFB1 (DMSO corrected) at 48h" = "((Treat_EBVAFB1_Pos + `Treat_EBVAFB1_Pos:as.factor(Time)48`) - (Treat_EBVDMSO_Pos + `Treat_EBVDMSO_Pos:as.factor(Time)48`)) - ((Treat_EBVAFB1_Neg + `Treat_EBVAFB1_Neg:as.factor(Time)48`) - Treat_EBVDMSO_Neg)",
    "AFB1+ (DMSO corrected) vs AFB1 (DMSO corrected) at 72h" = "((Treat_EBVAFB1_Pos + `Treat_EBVAFB1_Pos:as.factor(Time)72`) - (Treat_EBVDMSO_Pos + `Treat_EBVDMSO_Pos:as.factor(Time)72`)) - ((Treat_EBVAFB1_Neg + `Treat_EBVAFB1_Neg:as.factor(Time)72`) - Treat_EBVDMSO_Neg)"
)
contrasts[["timeseries_linear"]] <- c(
    "DMSO+ vs. DMSO linear time effect"                                  = "Treat_EBVDMSO_Pos:Time - Treat_EBVDMSO_Neg:Time",
    "AFB1+ vs. DMSO+ linear time effect"                                 = "Treat_EBVAFB1_Pos:Time - Treat_EBVDMSO_Pos:Time",
    "AFB1 vs. DMSO linear time effect"                                   = "Treat_EBVAFB1_Neg:Time - Treat_EBVDMSO_Neg:Time",
    "AFB1+ (DMSO corrected) vs AFB1 (DMSO corrected) linear time effect" = "(Treat_EBVAFB1_Pos:Time - Treat_EBVDMSO_Pos:Time) - (Treat_EBVAFB1_Neg:Time - Treat_EBVDMSO_Neg:Time)"
)
contrasts[["timeseries_quadratic"]] <- c(
    "DMSO+ vs. DMSO linear time effect" = "`Treat_EBVDMSO_Pos:poly(Time, degree = 2)1` - `Treat_EBVDMSO_Neg:poly(Time, degree = 2)1`",
    "AFB1+ vs. DMSO+ linear time effect" = "`Treat_EBVAFB1_Pos:poly(Time, degree = 2)1` - `Treat_EBVDMSO_Pos:poly(Time, degree = 2)1`",
    "AFB1 vs. DMSO linear time effect" = "`Treat_EBVAFB1_Neg:poly(Time, degree = 2)1` - `Treat_EBVDMSO_Neg:poly(Time, degree = 2)1`",
    "AFB1+ (DMSO corrected) vs AFB1 (DMSO corrected) linear time effect" = "(`Treat_EBVAFB1_Pos:poly(Time, degree = 2)1` - `Treat_EBVDMSO_Pos:poly(Time, degree = 2)1`) - (`Treat_EBVAFB1_Neg:poly(Time, degree = 2)1` - `Treat_EBVDMSO_Neg:poly(Time, degree = 2)1`)",
    "DMSO+ vs. DMSO quadratic time effect" = "`Treat_EBVDMSO_Pos:poly(Time, degree = 2)2` - `Treat_EBVDMSO_Neg:poly(Time, degree = 2)2`",
    "AFB1+ vs. DMSO+ quadratic time effect" = "`Treat_EBVAFB1_Pos:poly(Time, degree = 2)2` - `Treat_EBVDMSO_Pos:poly(Time, degree = 2)2`",
    "AFB1 vs. DMSO quadratic time effect" = "`Treat_EBVAFB1_Neg:poly(Time, degree = 2)2` - `Treat_EBVDMSO_Neg:poly(Time, degree = 2)2`",
    "AFB1+ (DMSO corrected) vs AFB1 (DMSO corrected) quadratic time effect" = "(`Treat_EBVAFB1_Pos:poly(Time, degree = 2)2` - `Treat_EBVDMSO_Pos:poly(Time, degree = 2)2`) - (`Treat_EBVAFB1_Neg:poly(Time, degree = 2)2` - `Treat_EBVDMSO_Neg:poly(Time, degree = 2)2`)"
)
```

### Statistical testing

Use MSqRob2^[Goeminne, LudgerJ. E., Gevaert, K., & Clement, L. (2016). Peptide-level Robust Ridge Regression Improves Estimation, Sensitivity, and Specificity in Data-dependent Quantitative Label-free Shotgun Proteomics *. Molecular & Cellular Proteomics, 15(2), 657–668. https://doi.org/10/f78fjf] to fit robust linear mixed models for every protein, precursor, or PTM and
perform testing.

```{r}
#| warning: false
#| label: chunk-msqrob

ridge_regression <- TRUE # flag for ease-of-use
# construct the contrast matrix
contrasts_full <- list()
L <- list()
for (i in seq_along(formulas)) {
    if (ridge_regression) {
        # prepend "ridge" to model parameters names to match output from msqrob function
        parameterNames <- paste0("ridge", colnames(model_designs[[i]]$designmatrix))
        # also do the above for the contrasts using regex matches to model parameter names
        # first escape all special characters from parameters to prevent the later regex from not matching
        regex_safe_parnames <- colnames(model_designs[[i]]$designmatrix) %>%
            # longest param first to prevent matching substring e.g. interaction terms
            {
                .[order(nchar(.), ., decreasing = TRUE)]
            } %>%
            str_replace_all(r"---{([\[\]\\*?+(){}^$|.])}---", r"---{\\\1}---") %>%
            {
                paste0("(", paste0(., collapse = "|"), ")")
            }
        contrasts_full[[i]] <- str_replace_all(
            contrasts[[i]],
            regex_safe_parnames,
            "ridge\\1"
        )
    } else {
        parameterNames <- colnames(model_designs[[i]]$designmatrix)
        contrasts_full[[i]] <- contrasts[[i]]
    }
    L[[i]] <- makeContrast(sapply(contrasts_full[[i]], paste, "= 0"), parameterNames = parameterNames)
}
# lists to store dataframes
hypothesis_tests <- list()
signif_pval_thresh <- list()
for (viz_assay in c("co-extracts", "variantMB", "precursorMB", "ptmMB")) {
    for (i in seq_along(formulas)) {
        # model fitting and hypothesis tests using MSqRob2
        pe <- msqrob2::msqrob(
            object = pe,
            i = viz_assay,
            formula = formulas[[i]],
            modelColumnName = names(formulas)[[i]],
            robust = TRUE,
            maxitRob = 20,
            ridge = ridge_regression,
            overwrite = TRUE
        ) %>%
            hypothesisTest(
                i = viz_assay,
                contrast = L[[i]],
                modelColumn = names(formulas)[[i]],
                overwrite = TRUE
            )
        # extract hypothesis test results
    }
    hypothesis_tests[[viz_assay]] <- do.call(rbind, rowData(pe[[viz_assay]])[, unlist(contrasts_full)]) %>%
        as_tibble() %>%
        mutate(
            contrast = {
                sapply(contrasts, names) %>%
                    unlist(use.names = FALSE) %>%
                    rep(each = nrow(pe[[viz_assay]])) %>%
                    factor(levels = unlist(sapply(contrasts, names))[!duplicated(unlist(sapply(contrasts, names)))])
            },
            name = if (viz_assay == "precursorMB") {
                rep.int(rowData(pe[[viz_assay]])$precursor, length(unlist(contrasts)))
            } else {
                rep.int(rownames(pe[[viz_assay]]), length(unlist(contrasts)))
            },
            family = rep.int(rowData(pe[[viz_assay]])$family, length(unlist(contrasts))),
            formula = rep(names(formulas), times = sapply(contrasts, length) * nrow(pe[[viz_assay]]))
        )
    # calculate BH threshold for each contrast using (rank of first non-significant test
    # / total number of tests * FDR)
    signif_pval_thresh[[viz_assay]] <- hypothesis_tests[[viz_assay]] %>%
        group_by(contrast, formula) %>%
        summarise(BH_thresh = (sum(adjPval < 0.05, na.rm = TRUE) + 1) / n() * 0.05, .groups = "drop")
}
```

And present the significant differences for every contrast:

```{r}
#| label: chunk-volcanos
#| output: false

assay_names <- list(
    "co-extracts" = "All proteins",
    "variantMB" = "Histone variants",
    "precursorMB" = "Histone precursors",
    "ptmMB" = "Histone PTMs"
)

volcanos <- list()
volcano_layout <- list()
for (viz_assay in c("co-extracts", "variantMB", "precursorMB", "ptmMB")) {
    for (formula_name in names(formulas)) {
        # static plot
        volcanos[[viz_assay]][[formula_name]] <- hypothesis_tests[[viz_assay]] %>%
            filter(formula == formula_name) %>%
            ggplot(
                aes(
                    x = logFC,
                    y = -log10(pval),
                    color = adjPval < 0.05,
                    shape = if (viz_assay == "co-extracts") {
                        NULL
                    } else {
                        family
                    },
                    text = if (viz_assay == "co-extracts") {
                        name
                    } else {
                        paste(family, name, sep = "#")
                    }
                )
            ) +
            geom_point() +
            geom_hline(
                data = filter(signif_pval_thresh[[viz_assay]], formula == formula_name),
                aes(yintercept = -log10(BH_thresh)),
                linetype = "dashed",
                color = "#c44133ff"
            ) +
            geom_vline(xintercept = 0, linetype = "dashed", color = "#253544ff") +
            facet_wrap(vars(contrast)) +
            theme_bw() +
            theme(plot.title = element_text(face = "bold")) +
            scale_color_manual(values = alpha(c("#253544ff", "#c44133ff"))) +
            coord_cartesian(xlim = c(-max(abs(filter(hypothesis_tests[[viz_assay]], formula == formula_name)$logFC)), max(abs(filter(hypothesis_tests[[viz_assay]], formula == formula_name)$logFC))))
        # print(volcanos[[viz_assay]][[formula_name]] +
        #     labs(
        #         x = bquote(log[2](FC)),
        #         y = bquote(-log[10](Pval)),
        #         color = bquote(Pval[BH] < 0.05),
        #         shape = "Histone family",
        #         title = paste0("Model ", formula_name, ", ", assay_names[[viz_assay]])
        #     ))
        volcano_layout[[formula_name]] <- ggplot_build(volcanos[[viz_assay]][[formula_name]])$layout$layout # for output width/height
        ggsave(
            plot = volcanos[[viz_assay]][[formula_name]] +
                labs(
                    x = bquote(log[2](FC)),
                    y = bquote(-log[10](Pval)),
                    color = bquote(Pval[BH] < 0.05),
                    shape = "Histone family",
                    title = paste0("Model ", formula_name, ", ", assay_names[[viz_assay]])
                ),
            filename = paste0(
                "./out/floats/",
                str_replace_all(assay_names[[viz_assay]], fixed(" "), "_"),
                "_",
                formula_name,
                "_volcano.pdf"
            ), width = 40 + 50 * max(volcano_layout[[formula_name]]$COL),
            height = 60 * max(volcano_layout[[formula_name]]$ROW),
            units = "mm", dpi = 300
        )
    }
}
```

::: {.column-body-outset}

::: {.panel-tabset}

```{r}
#| results: asis
#| out-width: 100%

for (viz_assay in c("co-extracts", "variantMB", "precursorMB", "ptmMB")) {
    for (formula_name in names(formulas)) {
        # legend label
        if (viz_assay == "co-extracts") {
            legend_shape <- NULL
        } else {
            legend_shape <- "\nHistone family"
        }
        cat("#### ", paste0("Model ", formula_name, ", ", assay_names[[viz_assay]]), "\n\n") # quarto programmatic tabset
        # interactive plot with plotly
        volcanos[[viz_assay]][[formula_name]] %>%
            plotly::ggplotly(tooltip = c("text", "x", "y"), height = max(volcano_layout[[formula_name]]$ROW) * 250) %>%
            plotly::layout(legend = list(title = list(text = paste0("Pval<sub>BH</sub> < 0.05", legend_shape)))) %>%
            htmltools::tagList() %>%
            print()
        cat("\n\n") # quarto programmatic tabset
    }
}
```

:::

:::

Finally, we can write out the volcano plots and results

```{r}
#| label: chunk-results

# write the results from differential abundance/usage analysis
for (viz_assay in c("co-extracts", "variantMB", "precursorMB", "ptmMB")) {
    # export results tables
    hypothesis_tests[[viz_assay]] %>%
        arrange(pval) %>%
        group_by(formula, contrast) %>%
        mutate(within_formula_contrast_rank = order(order(pval))) %>%
        ungroup() %>%
        mutate(
            signif = adjPval < 0.05,
            across(where(is.numeric), ~ signif(., 3))
        ) %>%
        write_csv(file = paste0("./out/floats/contrast_", viz_assay, ".csv"))
}
archive::archive_write_dir("./out/other/contrasts.zip", "./out/floats/")
```

The results of the differential abundance analysis can be downloaded here:

{{< downloadthis ../out/other/contrasts.zip label="Download results" type=info >}}
```
