# Supplementary material for: Aflatoxin B1 induces subtle but coordinated histone modifications in Epstein-Barr virus infected and non-infected Burkitt lymphoma cells
Source: Environ Int. 2025 Oct;204:109813. doi: 10.1016/j.envint.2025.109813 (PMC12715419; doi:10.1016/j.envint.2025.109813)
Supplement: Supplementary Figures [file mmc2.pptx]

## Slide 1
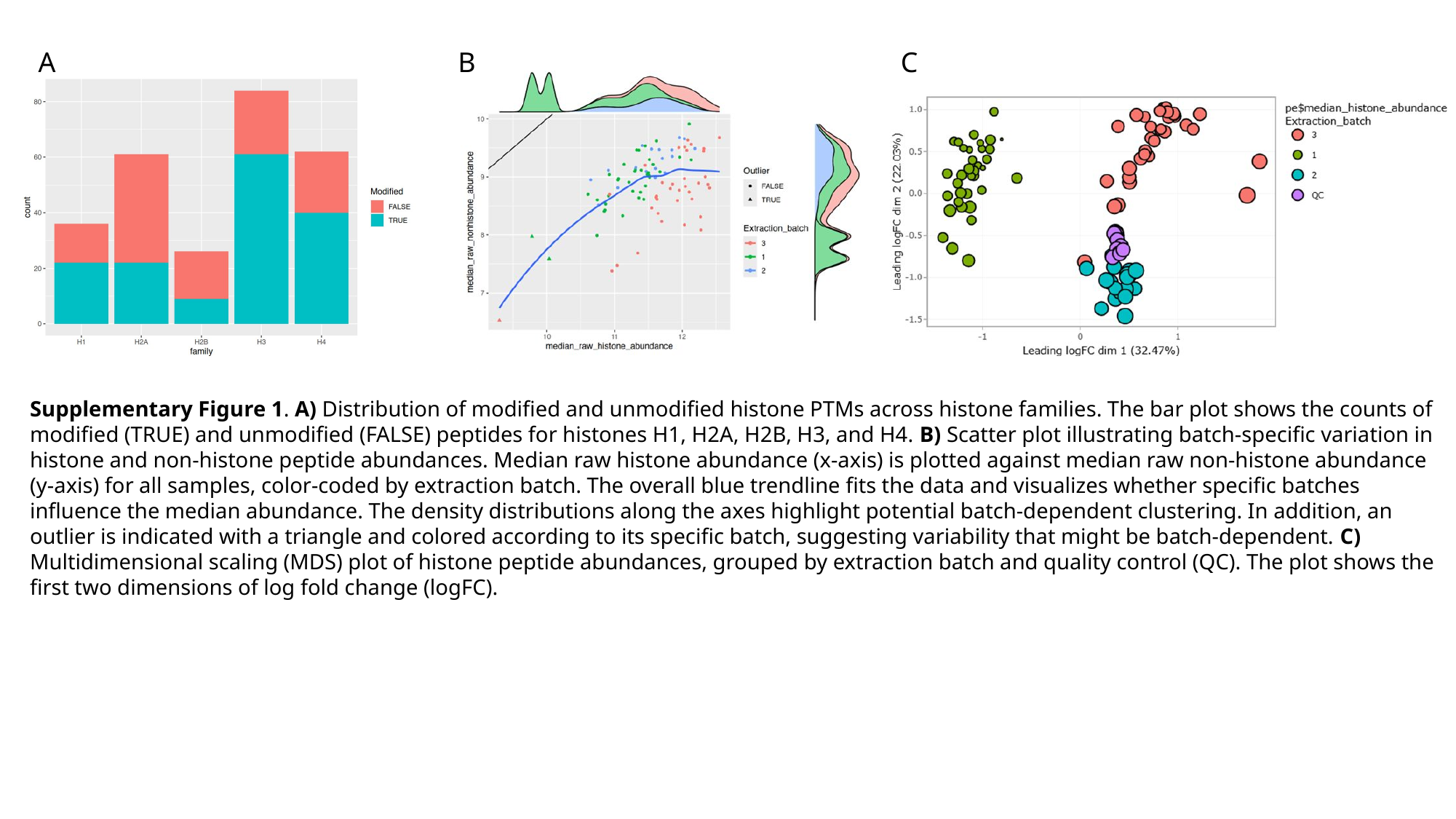

A
B
C
Supplementary Figure 1. A) Distribution of modified and unmodified histone PTMs across histone families. The bar plot shows the counts of modified (TRUE) and unmodified (FALSE) peptides for histones H1, H2A, H2B, H3, and H4. B) Scatter plot illustrating batch-specific variation in histone and non-histone peptide abundances. Median raw histone abundance (x-axis) is plotted against median raw non-histone abundance (y-axis) for all samples, color-coded by extraction batch. The overall blue trendline fits the data and visualizes whether specific batches influence the median abundance. The density distributions along the axes highlight potential batch-dependent clustering. In addition, an outlier is indicated with a triangle and colored according to its specific batch, suggesting variability that might be batch-dependent. C) Multidimensional scaling (MDS) plot of histone peptide abundances, grouped by extraction batch and quality control (QC). The plot shows the first two dimensions of log fold change (logFC).

## Slide 2
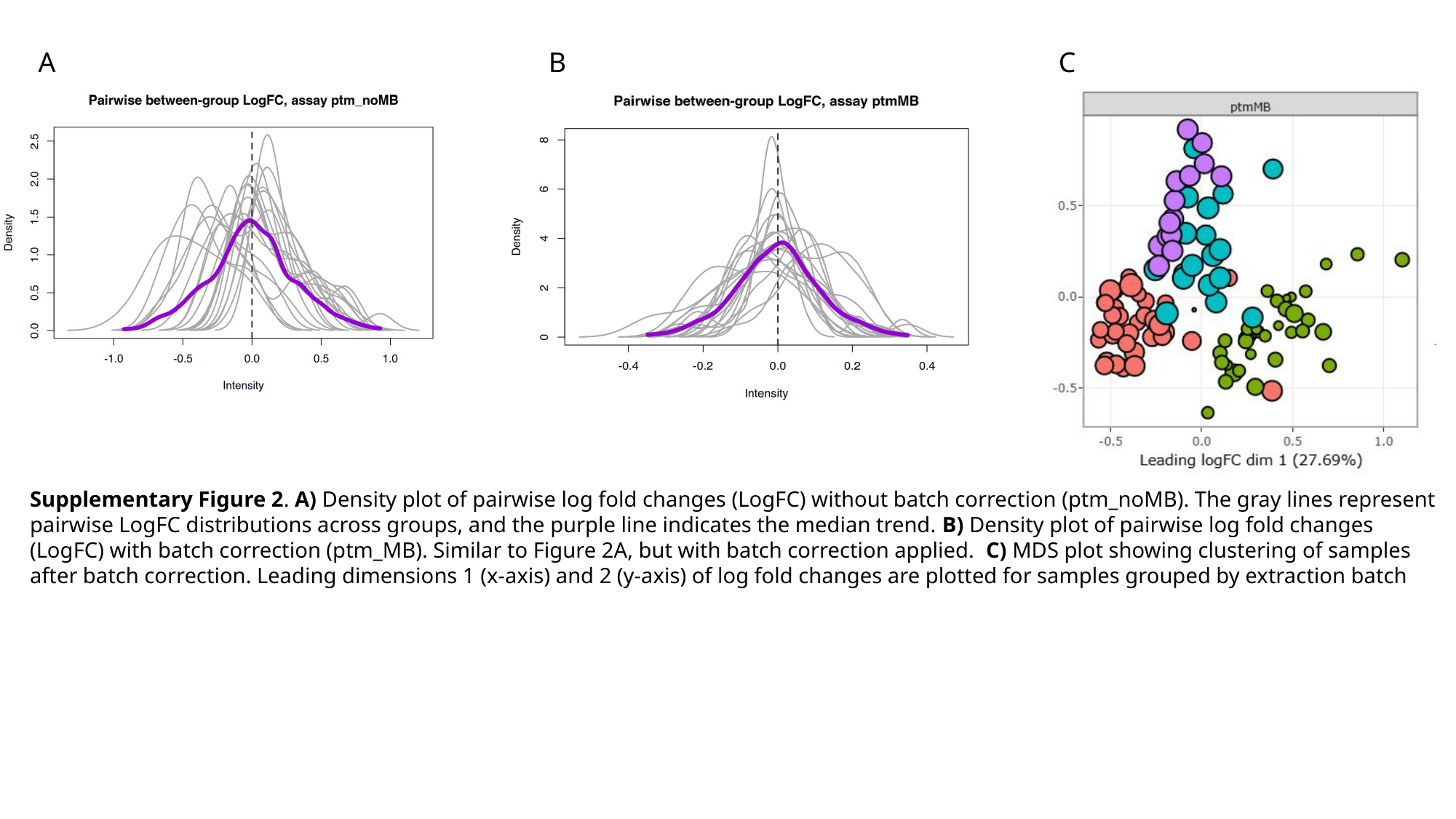

A
B
C
Supplementary Figure 2. A) Density plot of pairwise log fold changes (LogFC) without batch correction (ptm_noMB). The gray lines represent pairwise LogFC distributions across groups, and the purple line indicates the median trend. B) Density plot of pairwise log fold changes (LogFC) with batch correction (ptm_MB). Similar to Figure 2A, but with batch correction applied. C) MDS plot showing clustering of samples after batch correction. Leading dimensions 1 (x-axis) and 2 (y-axis) of log fold changes are plotted for samples grouped by extraction batch

## Slide 3
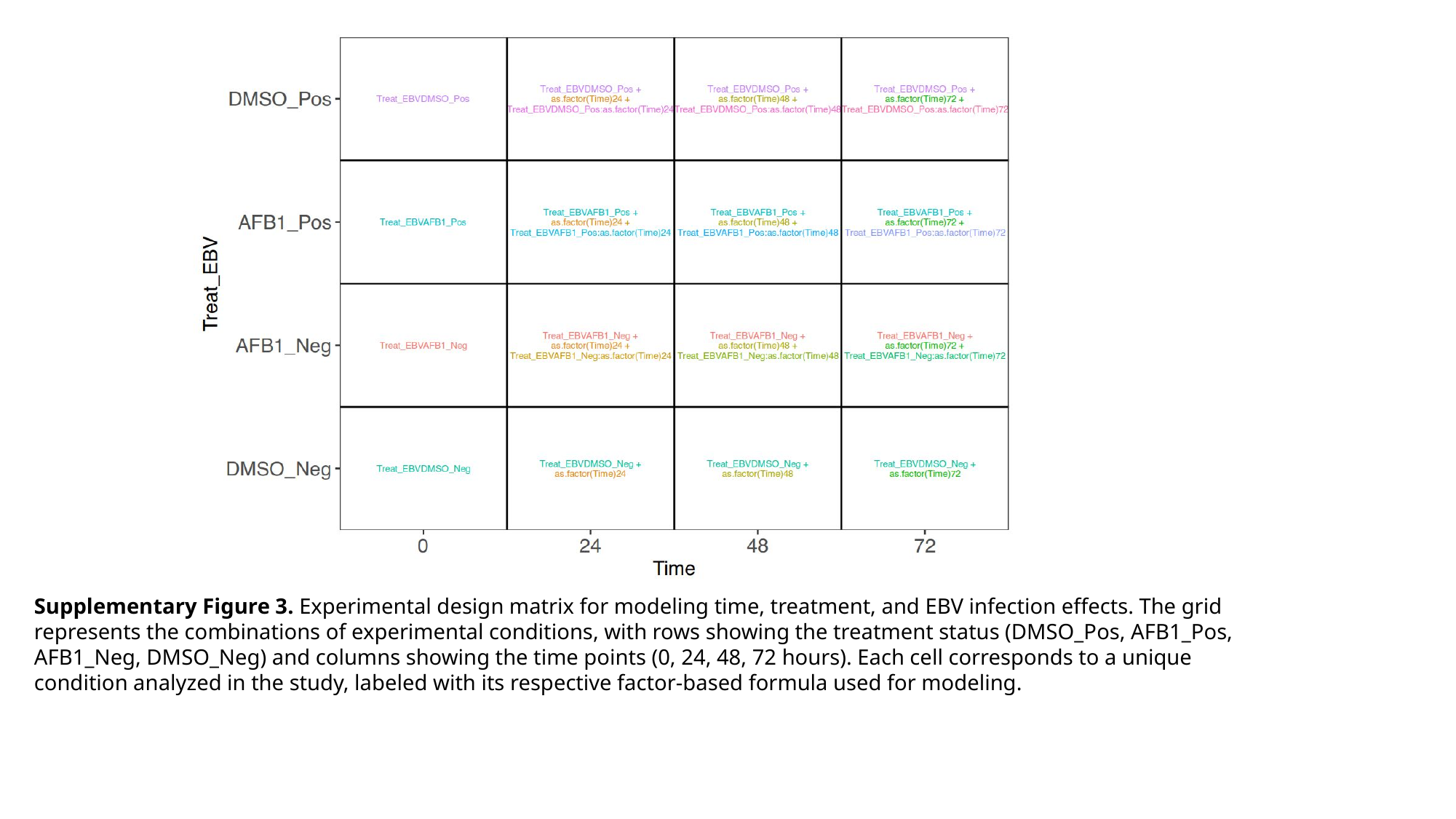

Supplementary Figure 3. Experimental design matrix for modeling time, treatment, and EBV infection effects. The grid represents the combinations of experimental conditions, with rows showing the treatment status (DMSO_Pos, AFB1_Pos, AFB1_Neg, DMSO_Neg) and columns showing the time points (0, 24, 48, 72 hours). Each cell corresponds to a unique condition analyzed in the study, labeled with its respective factor-based formula used for modeling.

## Slide 4
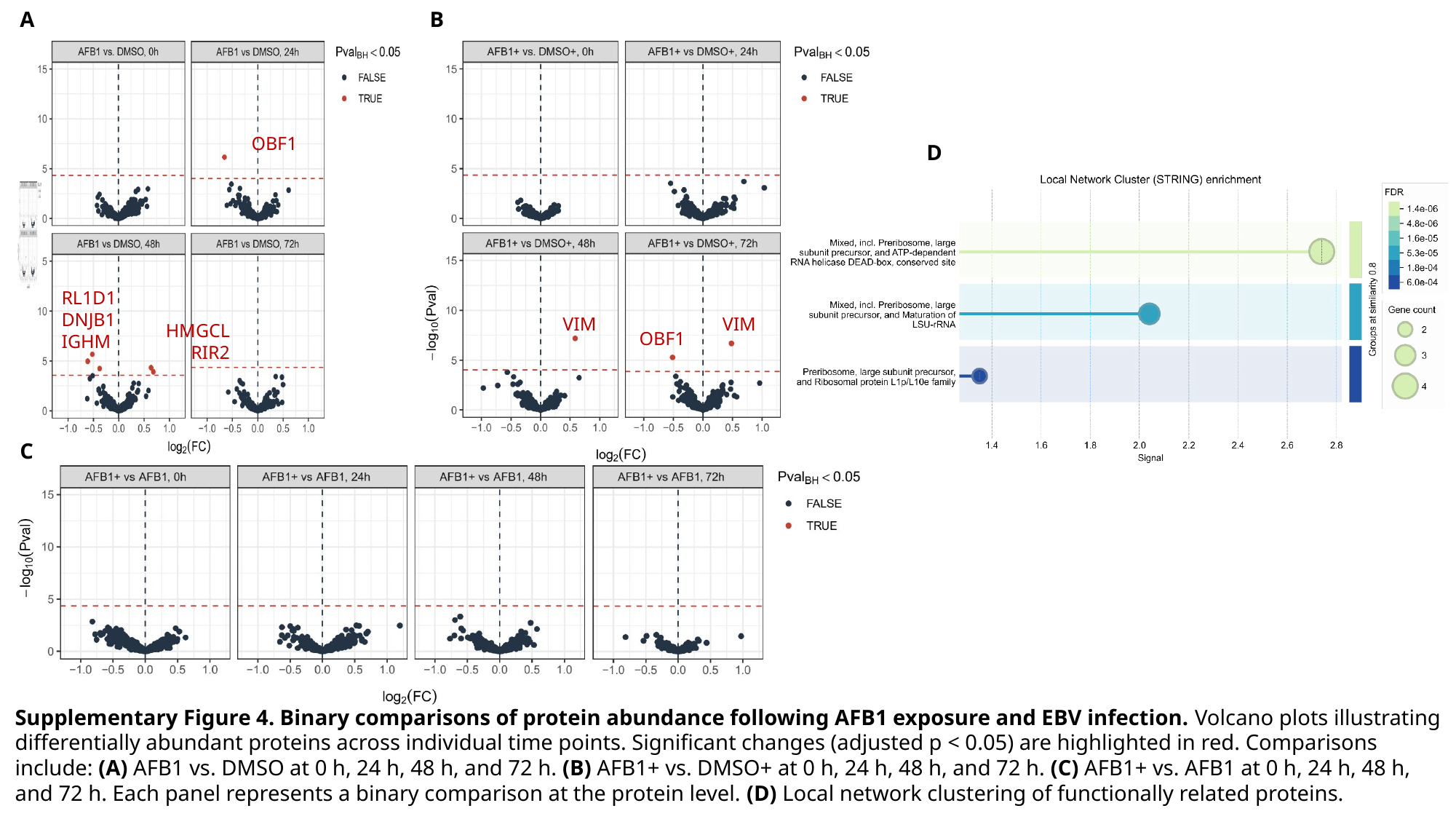

A
B
OBF1
RL1D1
DNJB1
IGHM
HMGCL
RIR2
VIM
VIM
OBF1
D
C
Supplementary Figure 4. Binary comparisons of protein abundance following AFB1 exposure and EBV infection. Volcano plots illustrating differentially abundant proteins across individual time points. Significant changes (adjusted p < 0.05) are highlighted in red. Comparisons include: (A) AFB1 vs. DMSO at 0 h, 24 h, 48 h, and 72 h. (B) AFB1+ vs. DMSO+ at 0 h, 24 h, 48 h, and 72 h. (C) AFB1+ vs. AFB1 at 0 h, 24 h, 48 h, and 72 h. Each panel represents a binary comparison at the protein level. (D) Local network clustering of functionally related proteins.

## Slide 5
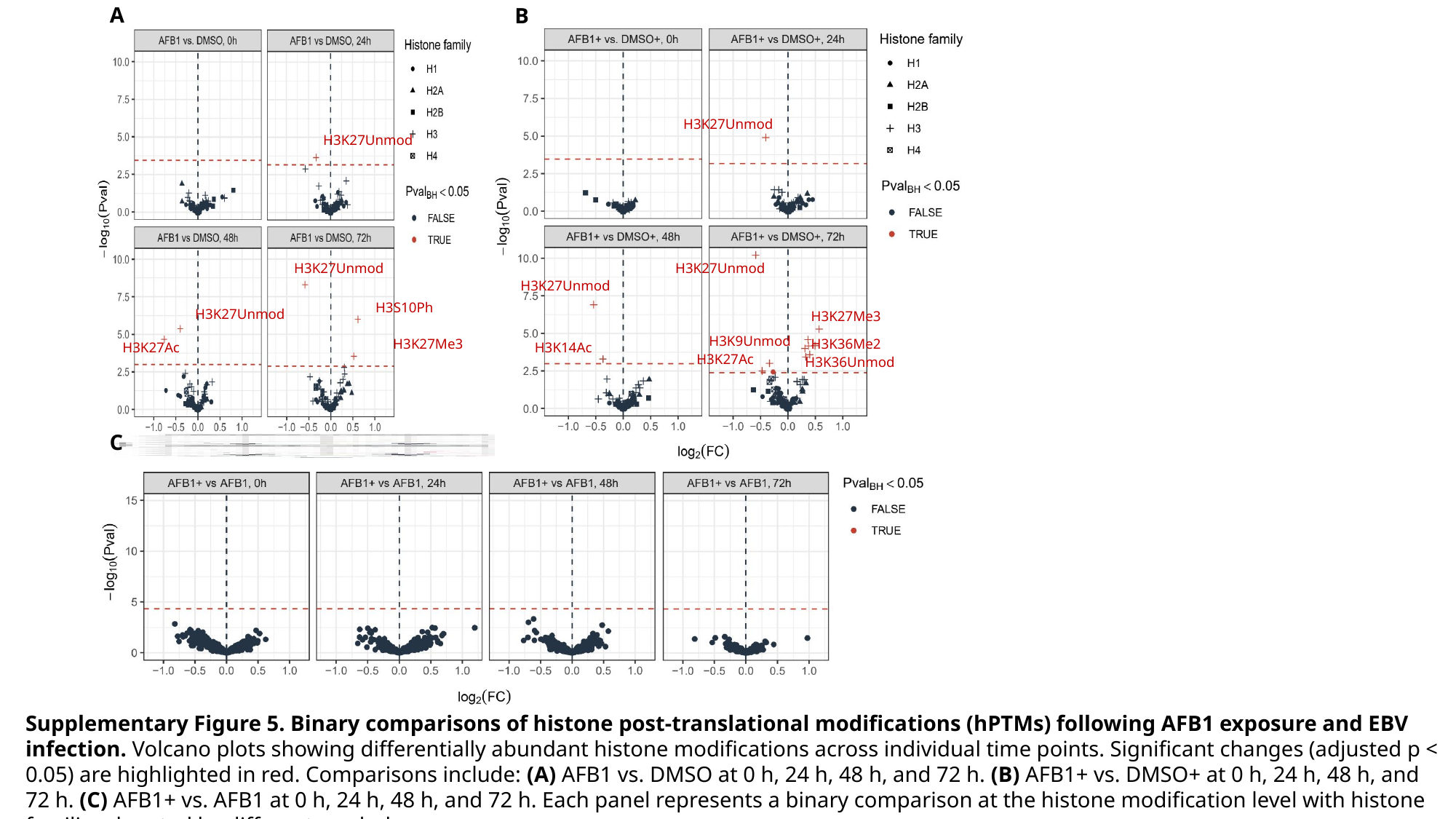

B
A
H3K27Unmod
H3K27Unmod
H3K27Unmod
H3K27Me3
H3K9Unmod
H3K36Me2
H3K14Ac
H3K27Ac
H3K36Unmod
H3K27Unmod
H3K27Unmod
H3S10Ph
H3K27Unmod
H3K27Me3
H3K27Ac
C
Supplementary Figure 5. Binary comparisons of histone post-translational modifications (hPTMs) following AFB1 exposure and EBV infection. Volcano plots showing differentially abundant histone modifications across individual time points. Significant changes (adjusted p < 0.05) are highlighted in red. Comparisons include: (A) AFB1 vs. DMSO at 0 h, 24 h, 48 h, and 72 h. (B) AFB1+ vs. DMSO+ at 0 h, 24 h, 48 h, and 72 h. (C) AFB1+ vs. AFB1 at 0 h, 24 h, 48 h, and 72 h. Each panel represents a binary comparison at the histone modification level with histone families denoted by different symbols.

## Slide 6
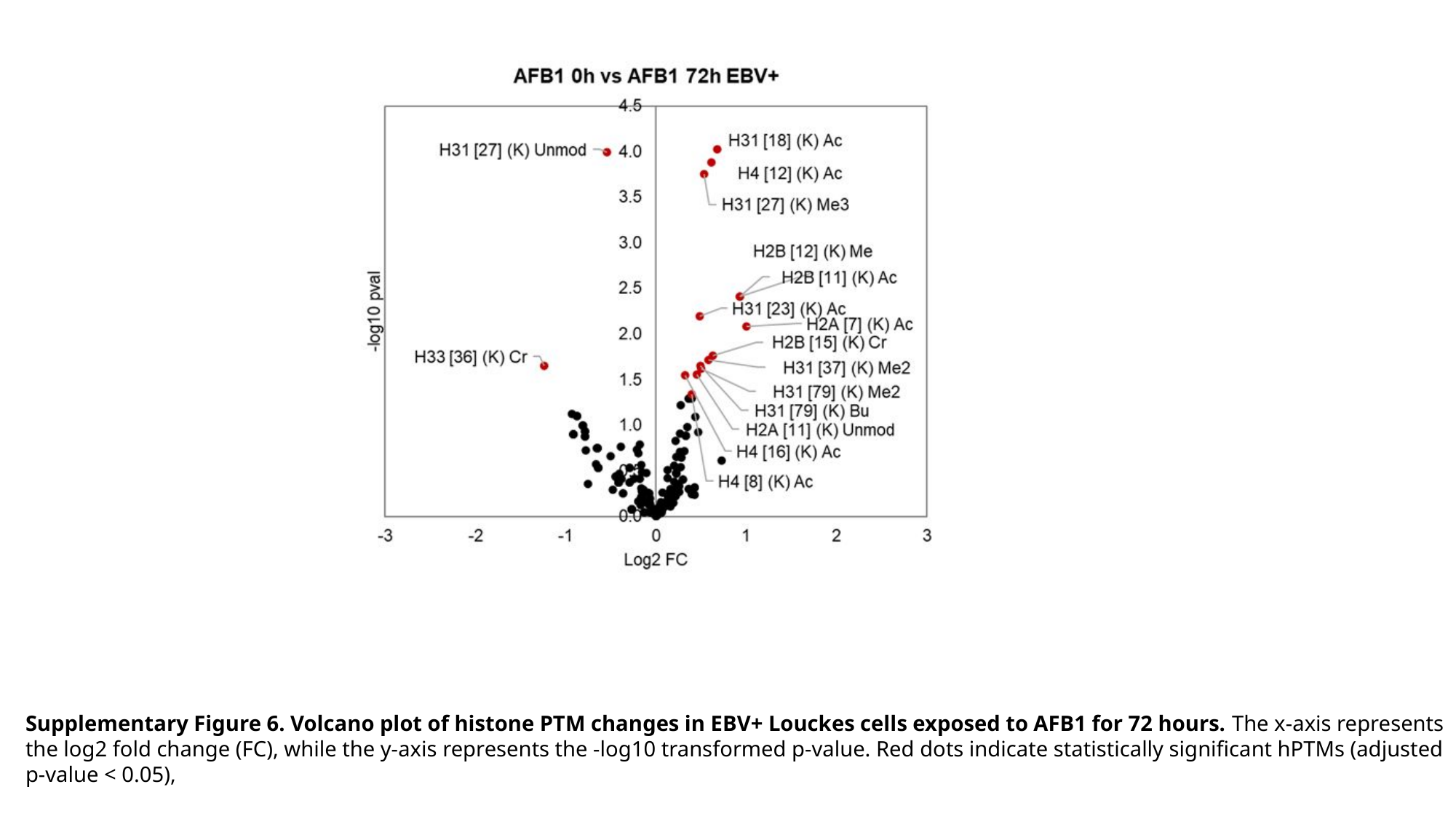

Supplementary Figure 6. Volcano plot of histone PTM changes in EBV+ Louckes cells exposed to AFB1 for 72 hours. The x-axis represents the log2 fold change (FC), while the y-axis represents the -log10 transformed p-value. Red dots indicate statistically significant hPTMs (adjusted p-value < 0.05),
